# Time Is of the Essence: Neural Codes, Synchronies, Oscillations, Architectures

**DOI:** 10.3389/fncom.2022.898829

**Published:** 2022-06-15

**Authors:** Peter Cariani, Janet M. Baker

**Affiliations:** ^1^Hearing Research Center, Boston University, Boston, MA, United States; ^2^Department of Otolaryngology–Head and Neck Surgery, Harvard Medical School, Boston, MA, United States; ^3^MIT Media Lab, Cambridge, MA, United States

**Keywords:** synchronies, oscillations, neural codes, temporal codes, radio communications, holographic memory, timing nets, neural networks

## Abstract

Time is of the essence in how neural codes, synchronies, and oscillations might function in encoding, representation, transmission, integration, storage, and retrieval of information in brains. This Hypothesis and Theory article examines observed and possible relations between codes, synchronies, oscillations, and types of neural networks they require. Toward reverse-engineering informational functions in brains, prospective, alternative neural architectures incorporating principles from radio modulation and demodulation, active reverberant circuits, distributed content-addressable memory, signal-signal time-domain correlation and convolution operations, spike-correlation-based holography, and self-organizing, autoencoding anticipatory systems are outlined. Synchronies and oscillations are thought to subserve many possible functions: sensation, perception, action, cognition, motivation, affect, memory, attention, anticipation, and imagination. These include direct involvement in coding attributes of events and objects through phase-locking as well as characteristic patterns of spike latency and oscillatory response. They are thought to be involved in segmentation and binding, working memory, attention, gating and routing of signals, temporal reset mechanisms, inter-regional coordination, time discretization, time-warping transformations, and support for temporal wave-interference based operations. A high level, partial taxonomy of neural codes consists of channel, temporal pattern, and spike latency codes. The functional roles of synchronies and oscillations in candidate neural codes, including oscillatory phase-offset codes, are outlined. Various forms of multiplexing neural signals are considered: time-division, frequency-division, code-division, oscillatory-phase, synchronized channels, oscillatory hierarchies, polychronous ensembles. An expandable, annotative neural spike train framework for encoding low- and high-level attributes of events and objects is proposed. Coding schemes require appropriate neural architectures for their interpretation. Time-delay, oscillatory, wave-interference, synfire chain, polychronous, and neural timing networks are discussed. Some novel concepts for formulating an alternative, more time-centric theory of brain function are discussed. As in radio communication systems, brains can be regarded as networks of dynamic, adaptive transceivers that broadcast and selectively receive multiplexed temporally-patterned pulse signals. These signals enable complex signal interactions that select, reinforce, and bind common subpatterns and create emergent lower dimensional signals that propagate through spreading activation interference networks. If memory traces share the same kind of temporal pattern forms as do active neuronal representations, then distributed, holograph-like content-addressable memories are made possible via temporal pattern resonances.

## 1. Introduction

The primary aim of this Hypothesis and Theory paper is to explore possible functional roles that neuronal oscillations, synchronies, and other temporal patternings of spikes might play in local and global neuronal circuits. Here we focus on their possible relations to neural codes. Finally, we briefly present some putative design principles for an integrated neural architecture, analogous to radio communication systems.

### 1.1 Reverse-Engineering the Brain

**FIGURE 1:**
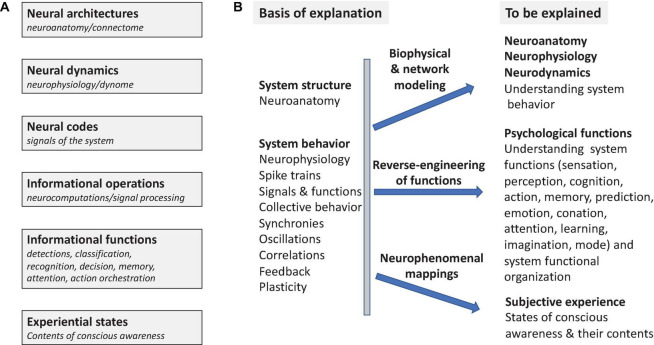
Systems Overview. The general problem of understanding how nervous systems work as informational and experience-producing systems. **(A)** Different aspects of the problem [cf. Marr’s levels of analysis (Marr, 1982)]. **(B)** What is to be explained (*explananda*) in what terms (*explanans*). Reverse-engineering seeks to elucidate operant functional principles in nervous systems using information processing models that account for mental functions. Neuro phenomenal mappings predict subjective experiences by elucidating the neural correlates of states of consciousness (NCCs) and of the contents of consciousness (NCCCs).

Reverse-engineering is the process of deducing the operating principles by which a complex system of unknown origin and/or structure achieves its functions. Understanding how the nervous system achieves its functions of controlling behavior is a large, long-term scientific reverse-engineering problem that encompasses several major aspects (Figure 1). This involves understanding the structure of the nervous system, individual and collective neuronal behavior, identifying the functional signals of the system (neural codes), characterizing its informational functions and the operations that subserve them, as well as understanding the neural basis of conscious awareness (Figure 1A). Ultimately functional and computational neuroscience seeks to account for how neuronal systems behave, to elucidate the neurocomputational processes by which they achieve the many informational functions that enable complex behaviors (Figure 1B). This goal also includes identification of the neuronal concomitants of states of awareness and their specific experiential contents.

### 1.2 The Neural Coding Problem

The neural coding problem entails identification and elucidation of which aspects of neural activity bear distinctions that subserve informational functions in the nervous system (Bullock, 1967; Perkell and Bullock, 1968; Perkell, 1970; Uttal, 1972; Cariani, 1995a; Stevens and Zador, 1995; Rieke et al., 1997; Nádasdy, 2000; Kumar et al., 2010). Whereas the *connectome* describes neuroanatomical interneural components and connections, and the *dynome* describes the dynamics of neuronal activity (Kopell et al., 2014), the realm of neural coding describes those aspects of dynome and the connectome that bear functional significance.

Here we take neural codes, in the strong sense of Watrous et al. (2015), to mean “that neural computation is causally driven by some configuration of spikes or extracellular signal, which implies that the brain is using this code to represent information.” Defined this way, neural codes are the functional “signals of the system.” They are neural activity differences “that make a difference.” Neural codes are thus recognized as codes on the basis of their “interpretation” by the rest of the system – how the nervous system uses them to achieve some function.

Neural representations that subserve perception, cognition, motivation, memory, affect, and the orchestration of action are all, in effect, neural coding schemes. On the sensory side, these representations are primarily generated by the action of external stimuli on sensory systems, whereas on the motor side, motor program representations for coordinated action are produced by adaptive internal pattern generation processes guided by sensory feedback and reward. Neural representations associated with drive states important for homeostasis and survival are presumably more likely to be based on dedicated circuits and coding schemes. Between sensory and motor systems lie neuronal assemblies and signals that self-organize from the internal informational dynamics of coordinating perception and action in service of anticipatory prediction and drive reduction.

Being part of a coherent, interpretable neural coding scheme separates those spikes that change internal functional states and external behaviors from those that don’t – not every spike need be interpretable by the system. Until evidence establishes that a given neuronal activity pattern exists and is linked to a particular function, prospective coding schemes are regarded as “candidate codes.”

The functional definition carries with it additional constraints. There must be some reliable mechanism whereby a possible code can be interpreted (read out) by the rest of the system (Perkell and Bullock, 1968; Kumar et al., 2010) and it must be capable of being integrated with codes for other attributes. If the distinctions it conveys can be retained in memory, then the original code or its transformations must also be compatible with available memory mechanisms.

### 1.3 Proposed Functional Roles of Synchronies and Oscillations

Following the discovery of brain rhythms less than century ago, many hypotheses concerning possible functions of neural synchronies and oscillations have been proposed and debated (Walter, 1959a,b; John, 1967b; Thatcher and John, 1977; Basar, 1988; Buzsáki, 2006; Nunez and Srinivasan, 2006; Uhlhaas et al., 2009; Klimesch, 2012; Singer, 2018, 2021). These include:

• *Neural coding of specific attributes.* Phase-locked synchronizations of spikes to external stimuli that precisely and robustly encode perceptual attributes can be found in almost every sensory modality, including audition, vision, mechanoreception, proprioception, and electroreception (Mountcastle, 1967; Carr, 1993; Cariani, 2001b; Lestienne, 2001). Stimulus intensity can be encoded in spike timings re: stimulus onset (Heil, 2004) and gamma cycle phases (Vinck et al., 2010). An extensive literature points to gamma-theta phase encoding of spatial place information in the hippocampus (Skaggs et al., 1996; Lisman, 2005; Lisman and Jensen, 2013).
• *Segmentation and binding processes*. Synchronies and oscillations have been proposed as general mechanisms for temporal grouping of events as well as the grouping of attributes associated with separate objects and events (Singer, 1999; Engel and Singer, 2001). Neural networks that bind through synchrony (Shastri and Ajjanagadde, 1993), common oscillatory frequency (Baldi and Meir, 1990) or phase (Klimesch et al., 2010), or common temporal patterns have been proposed (Reitboeck et al., 1988; Cariani, 2015).
• *Coupling/decoupling of neuronal subpopulations and regions by common timing (synchrony), oscillatory frequency, or common time patterns. Coupling through coherence* (CTC), in which common oscillatory frequencies and inter-regional synchronies may serve to couple or decouple different brain regions in a task-specific manner (Fries, 2015), is a leading general hypothesis. Flexible coupling of oscillators or neuronal integration in global circuits (Miller, 2013). Recently, a role for the cerebellum in coordinating inter-cortical communications through oscillatory control has been suggested (McAfee et al., 2021). Coherent coupling would also be critical for any heterodyne-like neural mechanisms (§ 8.1.3).
• *Support for memory.* Interactions between alpha, beta, and gamma in different cortical layers appear to mediate volitional retention of items in working memory (Miller et al., 2018). Gamma, theta, and delta oscillations are associated with memory consolidation operations in cortex, hippocampus, and striatum (Buzsáki and Moser, 2013; Headley and Paré, 2017).
• *Temporal scaffolding*. Hierarchies of nested oscillatory processes triggered by common events potentially provide a temporal framework for multi-level representations (O’Connell et al., 2015), such as integrating speech sequences at phonetic, syllabic, lexical, phrasal, and sentential levels (Shamir et al., 2009; Ding et al., 2016; Rimmele et al., 2021).
• *Temporal processing windows.* The relative timing of the presentation of parts of an object can determine which perceptual attributes are combined or separated. Loudness summation, how close together in time two identical sounds must be presented in order for their perceived intensities to sum is a primary example. Likewise, there are temporal integration windows for perceptual attributes in every modality and temporal windows for chunking sequences of perceptual events in time to form phrase structures, as in music and speech (Bregman, 1990; Snyder, 2000; Ding et al., 2016). Endogenous and externally-triggered oscillations may subserve these neuronal temporal integration processes.
• *Multiplexing of neuronal signals* in time-, frequency-, and temporal pattern-domains (§ 5.6).
• *Attentional gating, routing, amplification, and suppression mechanisms.* Oscillatory neuronal activity in particular frequency bands appear to support attentional processes that enhance immediately relevant information and/or suppress what is currently irrelevant (Klimesch, 2012; O’Connell et al., 2015). “Predictive routing” is mediated by characteristic layer-specific oscillations (Bastos et al., 2020). Via fast, precisely timed basal ganglia circuits (Pouzzner, 2020; Oberto et al., 2022), oscillations may be involved in gating inputs to cortical areas by intermittently disinhibiting thalamic inputs, thereby implementing temporal processing windows.
• *Time discretization and temporal ordering.* Sequences of gamma cycles (Uhlhaas et al., 2009) may serve to temporal discretize neuronal responses in order to convert continuous time patterns to discrete ordinal sequences that are invariant to changes in tempo (Shamir et al., 2009).
• *Reduction of spike jitter by suppressing membrane noise* (Schaefer et al., 2006).
• *Signal cancelation* via synchronized excitation and inhibition, desynchronizations or interference of competing oscillations. A striking example of signal cancelation is the phenomenon of binaural masking level difference (BMLD) where a tone rendered inaudible by masking noise is presented in one ear. If one then concurrently presents the same noise without the tone in the other ear, the tone is unmasked (by up to 15 dB) – subjectively it now pops out of the noise (Durlach and Colburn, 1978). The underlying neural cancelation mechanism is thought to involve matched excitatory and inhibitory binaural brainstem inputs that are phase-locked (synchronized) to the acoustic input common to both ears, i.e., the noise. There is an analogous unmasking effect in binocular vision (Henning and Hertz, 1973). In terms of the radio metaphor (§8.1), signals on particular oscillatory carriers as well as other oscillatory functions could conceivably be jammed by interfering oscillations with nearby frequencies or with phase-opposite oscillations of the similar frequency.
• *Emergence of new oscillatory frequencies* for increasing the dimensionality of neural signal spaces (§ 8).
• *Coupling to oscillatory bodily rhythms* (Klimesch, 2018), such as heartbeat, breathing, visceral processes, and circadian rhythms.

#### 1.3.1 Oscillatory Frequency

Although specific oscillatory frequencies vary considerably, there is strong evidence that different frequency ranges, denoted by Greek letters, are associated with different general functional roles. Below are some current notions of correspondences of oscillatory frequencies with information processing functions:

• *Gamma* (>30 Hz) oscillations are widely observed in cerebral cortex and hippocampus, especially in olfactory, visual, and auditory cortex. Gamma rhythms are generally associated with increased neural cortical activity related to selective sampling and coding of incoming inputs and outgoing actions. (Fries et al., 2007; Buzsáki and Wang, 2012; Lisman and Jensen, 2013; Cannon et al., 2014). Gamma frequencies and gamma power almost invariably increase under attention. In many cases gamma co-occurs with theta rhythms.
• *40 Hz gamma.* Specific evoked and induced gamma rhythms near 40 Hz are observed in auditory and visual regions, as well as hippocampus, cerebellum, and elsewhere (Galambos, 1992; Pastor et al., 2002). The 40 Hz “steady-state response” (SSR) has been used to “frequency tag” neural responses to other stimuli (Patel and Balaban, 2001b, a). Use of 40 Hz electrical, magnetic, photic, auditory, and vibrotactile stimulation is also actively being explored in therapeutic neurological contexts (Haller et al., 2020; Liu et al., 2021; Zhang et al., 2021; Mosabbir et al., 2022).
• *Beta* (∼15–30 Hz) oscillations are thought to be related to dynamic formation of flexible ensembles, such as working memory functions (Miller et al., 2018).
• *Alpha rhythms* (∼8–15 Hz) are thought to be involved with functional inhibition, i.e., the suppression of neuronal activity that is not currently relevant to current goals (tasks) (Basar, 2012). “Alpha oscillations are used as an electrophysiological indicator of vigilance and arousal, attenuating during targeted cognitive activity and strengthening while the brain is unoccupied by specific mental tasks and devoid of significant sensory input, particularly visual input. (Difrancesco et al., 2008).” Increased cortical extent and duration of alpha rhythms, but not alpha power, is correlated with transitions to acoustic parameters related to preferred spatial hearing percepts (Ando, 2009).
• *Theta rhythms* (4–10 Hz) are thought to be related to selective sampling of incoming information.
• *Coupled Theta-Gamma* rhythms are rhythms that may interact (“cross-frequency coupling”) such that they are initiated or phase-reset by a common triggering event to produce a nested hierarchy of oscillations (Canolty et al., 2006; Buzsáki and Wang, 2012; Lisman and Jensen, 2013). They are also thought be involved with organizing working memory (Chaieb et al., 2015; Reinhart and Nguyen, 2019).
• *Delta rhythms* (0.5–4 Hz) are prominent during sleep. They also may play a role in the temporal segmentation of incoming streams of events, such as speech (Rimmele et al., 2021).

#### 1.3.2 Oscillatory Power, Duration, Timing/Phase, Extent

In addition to oscillatory frequency, other potentially relevant parameters of oscillatory responses are: oscillatory power, temporal duration, oscillatory timing/phase, and spatial extent. Analogous parameters apply to neural synchronies as well: synchrony strength, duration, timing, and neural extent. *Oscillatory power* reflects the magnitude of the oscillatory frequency component relative to other components. The *temporal duration* of an oscillation wise a measure of how long the oscillation persists. Transient, oscillatory bursts can be quite short, from 1 to 3 cycles (Feingold et al., 2015), whereas sustained oscillations can persist for many cycles. *Effective duration* is a measure that quantifies the duration of coherent oscillations that is based on the decay of the envelope of the autocorrelation function as lag time increases (Ando, 2009; Ando and Cariani, 2014). Phase relations between multiple oscillations depend on their relative timings. Lastly, the *spatial extent* of an oscillatory pattern over brain regions is an indication of how widespread is the neural activation pattern. Different kinds of time-frequency representations may be appropriate for analyzing different aspects of oscillations (Bârzan et al., 2022).

Some of these parameters, such as the effective duration and cortical extent of alpha rhythms, are more correlated with listener-preferred concert hall architectural acoustic parameters, than more traditional measures such as alpha power (Ando, 2009; Ando and Cariani, 2014). Precise timing of oscillatory bursts may be critical for motor functions (Feingold et al., 2015).

Phase alignment is a form of synchronization. Phase relations between different oscillations in different brain regions may well be critical for their coordination (Sauseng and Klimesch, 2008). Phase resets may also be critical for neural coding (§5.4) as well as for synchronization of functionally-related neural populations (Klimesch et al., 2009). The relationships between oscillations and evoked neural activity patterns, including ongoing, induced and triggered oscillations, are complex (Sauseng et al., 2007; Klimesch et al., 2009).

Most of the proposed oscillatory functions involve information processing operations – how the brain processes neural signals – rather than representational functions – how the contents of those signals (attributes and specific distinctions of objects and events) are encoded. Arguably, once the neural correlates of information processing operations are firmly understood, then the scientific focus will then shift to problems related to neural coding (representation) and informational organization (scene analysis, binding and segmentation, and composition).

### 1.4 Causality, Correlation, Obligatory, Facilitative, and Tangential Roles

The nature of causality and time itself have long been contemplated and debated by philosophers and scientists (Reichenbach, 1956). In the neurosciences there have been perennial debates surrounding the neural causation of mental functions (Kim, 2011; Rolls, 2021). Whether oscillations and synchronies play causal roles in neural informational functions or whether they are correlative by-products of other processes that subserve these functions has been an abiding question in neuroscience (Sauseng and Klimesch, 2008). For example, one reviewer raised the thorny question of whether inter-regional synchronies might be byproducts of functional informational coupling rather than its causes.

How is a causal functional relation distinguished from a merely correlative one (Papineau, 1991)? Within neuroscience contexts, causal efficacy can be reasonably ascribed to some neural mechanism or process when its action reliably brings about some subsequent change in internal state or behavior, whereas its inactivation does not. If the relation is causal, the correlation between cause (activation of the neural mechanism) and effect is unity, whereas if other necessary factors are also involved, such that the correlation is substantially below unity, one has a correlative relationship. As correlations weaken, it becomes successively more difficult to ascribe causation. A strong correlation should lead investigation into possible underlying chains of neural events that might explain the causal relationship, at the same time discovering and ruling out possible common causes and “spurious correlations.”

There are also “interventionist” strategies for isolating causal chains (Woodward, 2008). Although current techniques of selective, reversible neuronal activation, stimulation, and pharmacological modulation are much more refined, the methodology is similar to classical lesion studies that analyzed loss-of-functions, with many of the advantages and pitfalls of such studies (Vaidya et al., 2019).

If there is but one underlying neural mechanism, then disrupting that mechanism should be sufficient to impair or abolish the functions it subserves. However, in the case of neural oscillations and synchronies, it may be difficult to rule out their complete abolition in local ensembles. Depending on the nature of the intervention, abolishing oscillations and/or synchronies might also disrupt other neuronal responses that could be necessary for the function at hand, leading to a potentially false conclusion of their causal efficacy. The existence of multiple parallel neural pathways that can realize partial function also can make establishing the causal role of any one path more difficult. In such cases, to prove causal roles, all parallel causal paths must be first eliminated and then each individual path must be tested for restoration of function.

In terms of realizing informational functions, oscillations and synchronies may be necessary (obligatory), helpful (facilitative), or functionally superfluous (neither helpful nor harmful). At one extreme, neural coding of the attributes themselves might depend entirely on spike synchronies or population oscillations. Prime examples are neural codes in the auditory system that utilize precise phase-locking: encoding of sound direction through interaural time differences (§5.4), echolocation based on echo delays (§5.4), and interval codes for pitch perception that convey delays related to sound periodicities (§ 5.3). Other strong examples exist in electroreception, flutter-vibration, and vision. In these cases, there is no function in lieu of precise, phased-locked spike synchronies.

Where synchrony and oscillatory timing is obligatory in sensory and motor systems, their modification or abolition should significantly alter or abolish percepts and actions. For the most part, introducing external stimuli (clicks, flashes, and shocks) that disrupt or reset normal oscillatory responses or configuring stimuli to reduce synchronizations do not dramatically alter basic percepts. For example, abolishing oscillations in insect olfactory systems using picrotoxin impairs fine, but not coarse, odor discriminations (§5.4.5). On the other hand, it has been long recognized that synchronizing timings of stimulation with ongoing brain rhythms can elicit rather unexpected effects, such as streams of memories (Walter, 1959b), suggesting that these rhythms are bound up with memory traces. Recent work is revisiting this experimental approach of oscillation-synchronized stimulation (Herrmann et al., 2016; Hohn et al., 2019).

At the other extreme, neural codes might operate almost entirely independently of population-wide oscillations and synchronies, rendering their functions impervious to changes in relative timings of external stimuli. Between these two extremes, neural information processing may be modestly enhanced if stimuli are presented at a favorable phase of a neural population oscillation. Attentional enhancement of relevant task-relevant signals by suppressing irrelevant neural responses may not be strictly necessary for most perception, but it certainly can make a difference in challenging, near-threshold conditions.

### 1.5 Relations to Neural Codes

One aspect of synchronies and oscillations often left out of discussions is the precise nature of the neural codes that bear the attribute information being grouped, separated, enhanced, suppressed, gated, grouped, transmitted, or retained in memory. What are the specific attributes? What are the neuronal signals that are being processed in these various ways? What are the neural codes such that the contents of these signals are encoded in patterns of spikes?

With the exception of phase-locked temporal codes, for the most part, it is conventionally assumed that the attributes themselves and their values are represented by rate- and channel-coded feature detectors, i.e., which particular neurons respond with higher firing rates (“spiking frequencies”). There are many reasons to doubt this general assumption (Gautrais and Thorpe, 1998). In many systems neuronal behaviors do not comport with functional roles as narrowly selective, unitary feature detectors, such that it appears that multiple types of information may be multiplexed in the same spike trains (Nelken et al., 2008; Bizley and Walker, 2010). In the auditory system, as well as in other sensory modalities, rate codes degrade at high stimulus intensities, whereas perceptual discriminations (Weber fractions) improve and perceptual invariances are retained. There also exist neural coding alternatives to rate-channel coding that bear exploration and examination. Accordingly, here we focus on these other coding possibilities.

### 1.6 Enlarging Our Thinking

How we think about neural coding is critical for understanding the functional roles of synchronies and oscillations in the brain. The aims of this paper are twofold: to explore the roles that synchronies and oscillations might play vis-a-vis alternative neural codes and to suggest ideas that we believe might be useful in formulating an alternative functional framework.

As this is a concept paper rather than a review, we will be discussing relations of neural synchronies and oscillations *to possible* neural coding schemes. We will attempt to convey which coding schemes are already well supported by experimental evidence and which ones are more putative. Our aim here is heuristic, to facilitate broader thinking into what kinds of temporal relations might subserve brain functions in hopes of provoking deeper questions and more insightful experiments.

## 2. Time in the Brain

The psychologist Mari Reiss Jones called time “our lost dimension” (Jones, 1976). A great deal of evidence points to the importance of the timing and temporal patterning of spiking activity in neural information processing. Not only is the nervous system operating *within time*, i.e., within an external temporal framework of events, but the basic signals of the system themselves may be *of time*, i.e., they might be temporal in nature.

Time is change. We hold as a working hypothesis that the brain is a self-organizing system that organizes itself to understand the world and effectively act within it. It is autoencoding in the sense that it chooses its own features and organizes its own input representations. There appears to be little in the way of genetic, *a priori* internal labeling of specific objects and events. That is to say, on the basis of the correlation structure of perception and action, brains program themselves, adaptively organizing neural codes, representations, and operations in order to anticipate consequences and to choose and orchestrate effective action that satisfies internal goal imperatives. In current machine learning jargon, they are “autoencoders” that adaptively construct their own codes and features. The neuromechanics of such adaptive and plastic cybernetic goal-directed systems are being worked out (Buschman and Miller, 2014). In short, brains are purposive anticipatory correlation machines (Bubic et al., 2010).

**FIGURE 2:**
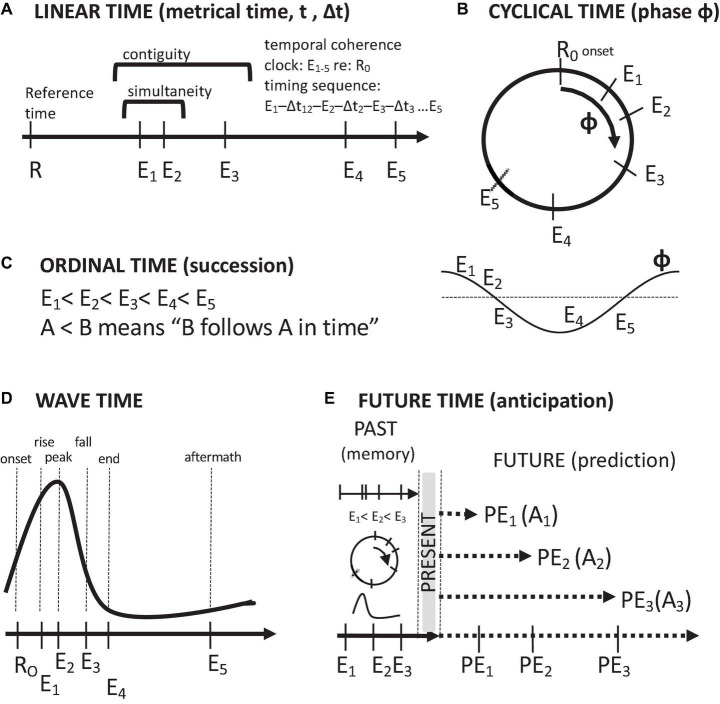
Descriptions of time and temporal relations. **(A)** Linear, metrical time. **(B)** Cyclical time. **(C)** Ordinal time (temporal order of succession). **(D)** Wave time. **(E)** Future time (anticipation/prediction on the basis of past memories and present events). Symbols: Arrows time axes, E event timings, R reference timings, PE predicted events, A set of event-associated attributes.

Thought about time in the brain follows five conceptions: linear time, ordinal time, cyclical time, wave time, and anticipatory, future time (Figure 2). These temporal orderings stem from how time is measured. Linear time involves the measurement of the timings of events in terms of a monotonically increasing metric, such as a clock value. Cyclical time involves labeling of the timings of events in terms of their measured phases of a repeating process. Ordinal time is assessed in terms of successions of events, i.e., temporal sequences of events. Wave time involves the timing of events with reference to a non-repeating event sequence of rise and fall. Future time involves the projection of the temporal order of remembered past events, along with their associated attributes, into the future. In this context, the purpose of remembering the past is to anticipate the future. Of course, remembering the past also has other, emotional uses, such as nostalgia and fond remembrance, as well as a source for motivation and resolve.

For the most part, discussions of neural synchronies tend to be couched in terms of temporal proximity relations between neural events and/or their adherence to reliable, linear timelines, whereas discussions of neural oscillations are inherently couched in terms of cyclical, recurrent timelines (Lestienne, 1999). There is some cross-over between these conceptions, as one can speak of the relative synchronization of the spikes of an individual neuron to population-wide oscillations and of recurring population-wide synchronies that constitute neural oscillations and brain rhythms.

## 3. Synchronies

Neural synchronies involve temporal relations between neuronal events on the level of individual neurons, ensembles and populations. The term “synchrony” can have several different meanings in different contexts (Lestienne, 2001). Some main senses of the term involve:

(1) *Externally-driven synchronization* (stimulus-locking or phase-locking). These consist of spikes and/or population responses that are time-locked to some external stimulus. They can also be driven by internal events, such as the synchronization of one population to another.
(2) *Entrainment* is phase-locked following of an external stimulus on a cycle by cycle basis (e.g., one spike per period of a pure tone), or, alternately, regular synchronization to a periodic stimulus (e.g., musical metrical entrainment in finger-tapping) or reliable following of an external rhythmic stimulus that need not be periodic (e.g., entrainment to speech envelopes).
(3) *Internally-driven synchronization* to internal neural events, such as synchronizations of different neuronal populations to each other (Singer, 2019).
(4) *Emergent synchronies* are synchronizations that appear over time.
(5) *Simultaneity* (zero-lag synchrony) – spiking or neuronal activity co-occurs within some specified, short time window.
(6) *Temporal contiguity –* (coarse temporal overlap). Neural activity co-occurs within some intermediate duration time window.
(7) *Temporal coherence* (temporally correlated spike timings, non-zero-lag synchronies) – neuronal responses reliably occur within some determinate set of temporal relations. Temporal coherence can also mean that temporal patterns of neuronal response share similarities.
(8) *Common temporal framework* (common timeline) neural events reliably occur within the same fixed linear, cyclical, or wave temporal framework.
(9) *Synchronous firing neural networks*, “synfire” chains and cycles (Abeles, 1982a, 1990, 2003) and polychronous networks, are neural architectures of delay paths and coincidence detectors (§7) whose elements fire when near-zero-lag synchronies from incoming spikes occur.

### 3.1 Externally-Driven Synchronization (Phase-Locking)

Perhaps the most pervasive form of neural synchrony involves stimulus-locked responses to external stimuli (e.g., “phase-locking” in auditory, visual, mechanoceptive, and electroceptive neurons) or internal events (e.g., phase-locking to cardiac, respiratory, or sniffing cycles to muscle stretches during movement); (Cariani, 1995b, 2001b; Lestienne, 1999, 2001).

Phase-locking enables temporal auto-correlation representations of stimulus time structure and temporal cross-correlation-based representations of stimulus direction (Cariani, 2001b; Lestienne, 2001). Both types of derived representations are extremely robust and, unlike other coding schemes, improve with higher stimulus intensities.

Phase-locking enables temporal coding of the time structure of incoming stimuli at all temporal levels from frequency and periodicity to slower modulations to rhythms. It can provide the basis for temporal pattern and spike time-of-arrival neural codes (§ 5). The neurogram shown in the Figure in (§ 5.3) illustrates the ubiquity of phase-locking in the auditory nerve. When spikes are time locked to a stimulus, times between spikes (interspike intervals) carry detailed information about its time structure. Distributions of “all-order” interspike intervals, i.e., between consecutive and non-consecutive spikes, produce a temporal neural representation of the stimulus autocorrelation function that can serve an alternate time-domain means of encoding the stimulus power spectrum.

Phase-locking also allows for localization of incoming stimuli by analyzing relative times-of-arrival at different body locations. Auditory localization in the horizontal plane by means of binaural temporal cross-correlation is most widely appreciated, but evidence for analogous localization mechanisms can be found in many other sensory domains as well, such as mechanoreception (von Békésy, 1967), electroception, vision (Carr, 1993), and olfaction (von Békésy, 1964).

As a generalization, one could hypothesize that wherever phase-locked information is available, it furnishes more precise and robust information than rate-channel coding. For example, in the auditory system, optimal use of spike timing information from auditory nerve fibers produces frequency acuities (Δ*f*/*f* pure tone Weber fractions) for pure tones that are 40-fold more precise than those using rate-place information, even at the low sound levels that are most favorable to rate-place codes (Siebert, 1968; Heinz et al., 2001a). At higher sound levels, where pitch acuities are best, spike timing information in the auditory nerve fiber population improves (Cariani, 1999) while rate-place representations break down due to saturation of firing rates and shifting best frequencies. Unlike pitch perception and temporal representations, rate-place codes fail when sound levels are roved (Heinz et al., 2001b). In the visual system, although thalamic units exhibit coarse, rate-based tuning to the spatial frequency of gratings moving at constant velocity, i.e., to different rates at which luminance is temporally modulated, spike timing information similarly yields more precise estimates of spatial frequency than does firing rate (Cariani, 2004).

Due to phase-locking and temporally patterned movements, perception and action can share common temporal codings. In addition to movements, there also exist pervasive cross-frequency couplings between neural populations and oscillatory rhythms generated by bodily organs (Klimesch, 2018). Neural phase-locking to rhythmic patterns of events (<10 Hz) exists in multiple modalities at the cortical level. Temporal correlates of both experienced and imagined auditory rhythms have been found (Nozaradan et al., 2011, 2013; Nozaradan, 2014). For the most part, rhythm has been modeled in terms of clocks, modulation-tuned neurons, and non-linear oscillators rather than as a direct temporal code. Direct temporal coding of rhythm means that incoming auditory temporal volley patterns that mirror the rhythmic structure can be shunted to motor regions to trigger muscle actions, such as finger-tapping and vocal mimicry. Likewise, when freely producing the same rhythmic actions, the motor system generates that rhythmic temporal pattern to coordinate the timing of groups of muscles. When muscles contract, mechanoreceptive afferents innervating stretch receptors produce spikes that are phase- locked to the ensuing movements. Thus these temporal patternings of body movements are in turn fed back into the brain such that the brain is continually bathed in the temporal structure of its actions. The external effects of patterned rhythmic action, such as drumming, create still other temporal pattern feedbacks in auditory, visual, and haptic modalities.

Thus, there may be a common neural language that underlies the temporal aspects of perception and action, such that perception and action can mutually inform each other in a direct manner. Keeping the neural signals in the time-domain and circulating in reverberating delay loops, as in recurrent neural timing nets (§7.5), permits common temporal pattern codes to be utilized in both perception and action. Hearing a musical rhythm provides a temporal scaffold for timing movements, and the timing of movements reinforces the perceived rhythmic structure of the music. Such percept-action correlations are likely generalized to many other modalities as well.

Synchronization can also occur internally, at neuronal, ensemble, population, and regional levels of organization. The synchronies can involve unitary events, oscillations, complex rhythmic patterns, or waves. Such synchronies can emerge over time, persist, or disappear. Most current thinking about inter-regional synchronies involves increased functional connectivity through facilitation of information transfer, e.g., Singer (2019).

### 3.2 Simultaneity and Temporal Contiguity

Response simultaneity is the co-occurrence of events in temporal proximity, i.e., at approximately the same time. Depending on criteria for temporal contiguity, i.e., what counts as “the same time,” co-occurrences of spiking events can range from temporally precise (<<1–20 ms zero- and near zero-lag synchronies) to coarse temporal overlaps of synchronies between different neuronal populations (20 – 500 ms coarse synchronies).

The issue of temporal overlap involves windows for summing the effects of successive spikes in single neurons and of volleys of spikes in neural populations. The notion of “firing rate” itself implies temporal contiguity between spikes, i.e., a temporal counting window that encompasses at least two spiking events.

Durations of coincidence windows are governed by a host of synaptic and membrane biophysical parameters as well as by types, numbers, efficacies and spatial distributions of neural inputs. Precisely timed inhibitory inputs can narrow these windows substantially (Ashida and Carr, 2011). Spike-timing-dependent plasticity (STDP) is also widely observed (Feldman, 2012; Markram et al., 2012), in which the effects of synchronized synaptic inputs on producing subsequent action potentials are facilitated, whereas those of unsynchronized inputs are depressed (Bi and Poo, 1998; Sjöström and Gerstner, 2010). In addition, action potentials produced by more synchronized synaptic inputs may have larger downstream effects on subsequent local networks (Zbili et al., 2020).

Different estimated durations of neural integration windows from spiking variability have led to discussions of whether cortical pyramidal cells should be seen primarily as integrators (long windows > 20 ms) or coincidence detectors (short windows < 5 ms) (Abeles, 1982b, 1994, 2003; Softky and Koch, 1993; Konig et al., 1996). Integrators imply rate-channel connectionist architectures, whereas coincidence detectors imply synfire chains, polychronous networks, wave interference networks, and neural timing nets, with mixtures of the two types implying time-delay neural networks (§ 7).

### 3.3 Temporal Coherence

Besides temporal contiguity (simultaneity), synchrony can also have the meaning of being part of a common temporal order. Events can be “synchronized with” other events if they occur at some regular, fixed delay relative to each other (delayed across-neuron synchrony) or to a common reference time (onset-referenced synchronies, typically a population-wide response to a stimulus onset or abrupt change). In the temporal order sense, although the various notes of the different instruments may occur at different times, the actions of a symphony orchestra are all synchronized to its conductor’s baton because they are all part of a common, ongoing temporal order. Alternately, the instruments in a MIDI score are all synchronized to the onset time of the entire score. Both temporal orders can serve as scaffolds for the coordination of neural activity. As discussed below, some neural codes, such as synchrony-place codes, depend on patterns of spike latencies relative to some common onset time, whereas other synchrony binding codes do not.

## 4. Oscillations

Whereas synchronies involve temporal relations between discrete neural events and linear timelines, oscillations involve recurrent temporal patterns of events that can serve either as common time references or as cyclical temporal scaffolds. Caution should be taken not to conflate the two concepts (Lestienne, 1999).

As with “synchrony,” the terms “oscillation,” “oscillatory behavior,” “oscillator,” and “oscillatory system” have multiple, but related, meanings. The most restrictive sense of oscillation involves an observable that periodically traverses a set of positional states around some central state. An oscillator is a postulated physical system, such as a pendulum, that manifests such regular regenerative, cyclic behavior. A second, more general sense of “oscillation” is any process that produces some regular cyclic sequence of events, be it with a fixed, characteristic period or not. Related to the idea of oscillation is the notion of resonance.

Resonance is the property of having a response, however, defined, that is greatest at some particular input frequency. Typically, this means a response of higher amplitude for particular driving frequencies of stimulation. Electrical resonances are found in individual neurons (Raymond and Lettvin, 1978; Hutcheon and Yarom, 2000). At the level of neuronal ensembles, resonance can also mean that a neural assembly responds differentially to different specific input patterns, such that it can manifest a “pattern resonance” or, if the resonance can be tuned through training, an “adaptive resonance” (Grossberg, 2021). Neurons can be regarded as oscillatory “integrate-and-resonate” instead of “integrate-and-fire” elements (Izhikevich, 2001), and resonances in neural oscillatory networks can switch behavioral modes (Greene, 1962). Neural timing nets (Cariani, 2001a, 2015), wave-interference (Heinz, 2004, 2010), and time-domain holography schemes (Longuet-Higgins, 1989) (§7, §8) raise the possibility of neural information processing based on “temporal pattern resonances.”

Individual neurons can be regarded as externally driven, non-linear oscillators that undergo cycles of action potential generation and recovery. Observed neuronal population dynamics also show cycles of activation, depression, and recovery that oscillate around resting states. Although neural oscillations are most commonly graphically depicted as sinusoids, and analyzed using frequency-domain Fourier descriptions, the time courses of their underlying biophysical processes need not be, and most often are not, sinusoidal (Nikolic et al., 2013).

Typically, “neural oscillations” or “brain rhythms” refer to observed aggregated, quasi-periodic responses of populations of neurons. Any temporal structure observed in these aggregated gross electrical potentials (EEG, evoked potentials) or magnetic fields (MEG) reflects neuronal dendritic and spiking activity that is synchronized across large neural populations.

As with “synchrony,” the term “oscillation” is used in several different, albeit related ways:

(1) *Endogenous oscillations* or *intrinsic rhythms* are “spontaneous” neural oscillations in the absence of driving stimulation (Walter, 1959a) that are commonly thought to reflect resting brain states or the natural resonances of neuronal population dynamics. A second sense of endogenous oscillation is “endogenous neural oscillations as rhythmic neural activity that originates from the brain, and is therefore also present in the absence of stimulus input” which allows for interactions of endogenous oscillations with evoked, stimulus-driven periodicities (Zoefel et al., 2018).
(2) *Stimulus-driven, evoked or facilitated oscillations* are oscillatory responses to driving stimulation. Gamma rhythms (30 Hz and above) are observed in response to stimulation, with higher intensities generally producing higher oscillatory frequencies (Buzsáki and Wang, 2012).
(3) *Stimulus-driven entrainments* are stimulus-locked responses to periodic and quasi-periodic stimuli (Nozaradan, 2014).
(4) *Stimulus-triggered oscillations* are oscillations that are initiated or phase-reset in response to a stimulus. The N100 response that is observed in auditory evoked electrical potentials and magnetic fields is an example (Pantev et al., 1991).
(5) *Induced oscillations* are “oscillations caused or modulated by stimuli or state changes that do not directly drive successive cycles” (Bullock, 1992).
(6) *Assimilated rhythms* are rhythmic response patterns that are acquired through training or electrical conditioning (John, 1967a,b; Morrell, 1967).
(7) *Emergent oscillations* are oscillations that develop over time.
(8) *Nested oscillations or oscillatory hierarchies* are oscillations of different frequencies whose phases are reset together. An example is the co-appearance of theta and gamma rhythms beginning in common phase in response to an acoustic onset (Pantev et al., 1991). Theta-gamma coupling in the hippocampus appears to be related to working memory (Lisman and Jensen, 2013).
(9) *Quasi-cyclical trajectories* in dynamical or data-derived phase spaces.
(10) *Adaptive oscillators* are systems that have dynamically controlled oscillatory elements (Hoppensteadt and Izhikevich, 2000). For example phase-locked loops can track periodic and quasi-periodic signals such as speech. Neural phase-locked loops have been proposed for converting temporal codes to rate codes (Ahissar et al., 1997) and for quasi-oscillatory tracking of speech (Shamir et al., 2009; Ghitza, 2011).
(11) *Standing and traveling spatial waves* are spatiotemporal patterns of neuronal excitation and recovery in local regions and on global scales (Nunez, 2000; Nunez and Srinivasan, 2006; Thorpe et al., 2007; Bhattacharya et al., 2022).
(12) *Oscillatory neural networks* are networks of coupled oscillators (e.g., Baldi and Meir, 1990; Hoppensteadt, 1997; Izhikevich, 1999).

## 5. Neural Codes

Possible functional significances of neuronal synchronies and oscillations have long been debated. The neural coding problem entails determining which aspects of neuronal activity convey distinctions that subserve informational functions (Mountcastle, 1967; Perkell and Bullock, 1968; Perkell, 1970; Uttal, 1972, 1973; Cariani, 1995b, 1997, 1998). From this perspective, the realm of neural codes involves a subset of the aforementioned *dynome*, and the question of whether oscillations are causal, correlative, or merely incidental to informational functions is fundamentally a question of their relations to neural coding.

Although neural coding includes both analog continuous electrical fluxes (local current flows and fields) and trains of discrete pulsatile spikes, for this discussion we adopt the basic working assumption that all information, if it is to be transmitted beyond its originating locale, will eventually be encoded in spike trains. Neural codes can operate on sequences and distributions of spikes at sub-neuron, neuron, ensemble, and population levels. Aggregate measures of neuronal activity, such as local field potentials, gross potentials, magnetic fields, and blood oxygenation levels can serve as windows on underlying neural codes even if what they measure may be provide correlates of, and not direct causal linkages to, informational functions.

Neural codes serve to encode information for all essential neuropsychological functionalities: sensation, perception, cognition, conation, emotion, short- and long-term memory, attention, learning, prediction, deliberation, modal control (wake-sleep) and bodily regulation. A further working assumption is that, while there may exist neural codes that are only localized to specific brain regions, there is a general neural coding framework that enables most/all kinds of information to be integrated within a common *lingua franca* in a manner loosely analogous to the genetic code.

**FIGURE 3:**
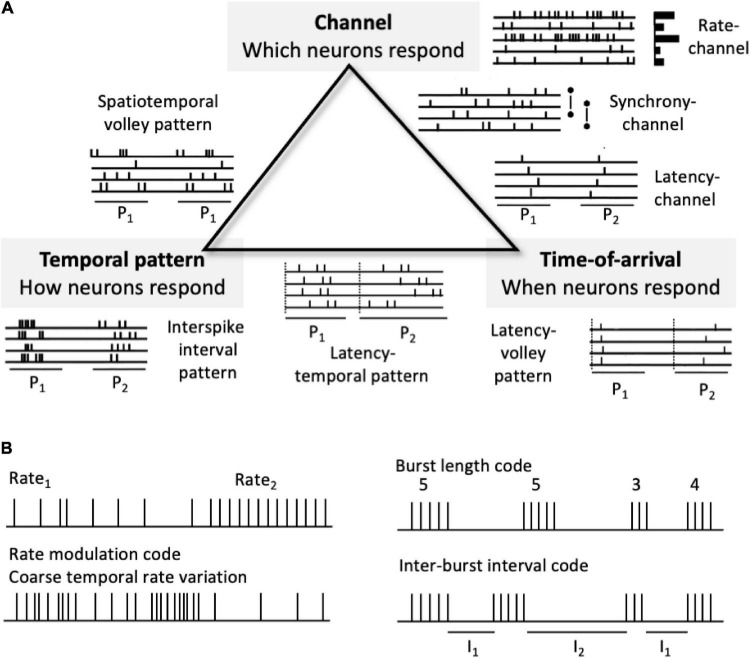
Neural pulse code schemes. **(A)** A partial taxonomy of types of neural codes. At the triangle vertices are pure coding types, whereas the edges indicate combinations of two different types. P_1_ and P_2_ indicate two different spike patterns in the idealized spike raster icons. **(B)** Examples of other codes. Top left. A simple rate-channel “doorbell” code. Bottom left. Rate modulation code or coarse “temporal” code. Right. Burst length (# spikes per burst or duration of burst) and inter-burst interval (I_1_ and I_2_) codes. Rate modulation and burst-based codes don’t fit neatly into the taxonomy.

Many kinds of neural pulse codes are possible, although a basic taxonomy can capture much of the space of possible neural codes (Figure 3). Here codes can be divided into those that depend on patterns of neural channel activations, temporal patterns of spikes, and relative spike timings across channels. Neural coding strategies can be combined, making the space of all conceivable pulse codes potentially quite complex. Neural pulse codes need to convey two different types of information: signal type (what attribute?) and signal value (what specific distinction within that attribute?). Signal type information involves the dimension or category of information being conveyed, such as sensory modality (e.g., visual, auditory, and olfactory) and the attribute within that modality (form, color, texture, pitch, loudness, location, and smell). Signal value involves a distinction within the type category, (which form, color, texture, pitch, loudness level, and apparent location).

In addition to conveying type and value distinctions, neural coding systems must also support organization of attributes amongst multiple objects and events (perceptual organization, Gestaltist grouping, segmentation and binding, scene analysis). In order to represent multiple objects occurring at the same time, each having its own coded attributes, some compositional, binding process is required for grouping together the coded attributes associated with each object. Such a mechanism is also needed for binding together the various attributes of events in memory.

Our working assumption is that brains are highly plastic, self-encoding systems that adaptively construct their own internal codes on the basis of correlational regularities in perception and action, many of which are temporal in nature. There are also neural mechanisms involved in selective task-related, attentional gating of neuronal signals that can amplify or suppress different types of incoming information or outgoing motor operations by facilitating or inhibiting particular neural channels (channel codes) or temporal patternings (temporal codes).

### 5.1 General Types of Neural Pulse Codes

A high level, partial taxonomy of types of neural codes (Figure 3A) includes codes based on channel-identity, temporal pattern, and response time-of-arrival (spike latency). Based on their encoding/decoding schemes, many, possible pulse codes can be divided into two broad types: channel-based codes and temporal codes. Channel codes rely on which particular neural channels are activated or distinguished such that information is conveyed via across-neuron patterns of response. The channels can be different individual neurons, ensembles, subpopulations, or populations. Here channel-identities convey signal types, whereas some other marking variable, such as average firing rate, latency, order, or variability conveys the value of that representational distinction. The taxonomy is by no means exhaustive. Figure 3B shows some plausible codes, such as those that depend on coarse temporal patternings of firing rates (including firing rate variability) and those that depend on bursts of spikes, that do not readily fit into this tripartite scheme.

Temporal codes, in contrast, rely on patterns of spike timings to convey signal types and informational distinctions within those types. Temporal codes can be divided into two basic subtypes: temporal pattern codes and spike latency or relative time-of-arrival codes. Temporal pattern codes are based on volley patterns of spikes, irrespective of their absolute spike times, whereas spike latency codes are based on spiking timing in relation to some time reference point, irrespective of the temporal patterns within. In terms of signals, temporal pattern codes depend on the internal form of the signals, whereas time-of-arrival codes depend on the relative timing of signals irrespective of their internal form. Because there is no precise neural representation of absolute time *per se*, all neuronal time is relative. However, temporal reference can be subserved by the timing of other neural responses, such as neurons, ensembles, subpopulations, or populations that respond with short latencies to onsets of external and internal events. Spike latencies are therefore temporal offsets from some other neural reference time marker.

Neural signal processing models commonly regard neurons as filters, i.e., elements whose responses are differentially sensitive to particular aspects of their inputs. For example, auditory nerve fibers are often modeled in terms of their firing rate responses to pure tones of different frequencies, i.e., as frequency-domain band-pass filters. In terms of neurons-as-filters metaphors, channel codes use the energies of filter outputs as indicators of channel activation, whereas temporal codes use the temporal patterning of or the timing of neural spike train output signals as indicators. In terms of feature detector metaphors, channel codes use elevated rates or response latencies in selective, feature-tuned channels to mark detection of specific features, whereas temporal codes encode the features in the time structure or timing of neural responses. In terms of signal processing, the primitives of the coding types lie in separate domains: channel codes operate in the “channel-domain,” whereas temporal codes operate in time- and time delay-domains. Codes involving oscillatory frequencies and their interactions lend themselves to frequency-domain descriptions.

Both channel and temporal codes permit vectorial representations. In channel codes, vector representations consist of profiles of channel activations, whereas in temporal codes, they consist of temporal pattern of spike timing profiles. For example, a cochlear rate-channel code conveys a vectorial representation of a stimulus power spectrum that consists of the firing rates of auditory nerve fibers that are selectively tuned to a particular range of pure tone frequencies. A temporal pattern code, such as an interspike interval code (§ 5.3) conveys a vectorial representation consisting of the distribution of interspike intervals, i.e., time delays between spikes. A first-spike latency code conveys a vectorial representation by means of distributions of first-response-times amongst channels. The temporal dispersion of first-spike times amongst channels, without consideration of which channels are associated with which spike latencies, is another potential means of encoding the intensity of a transient stimulus.

Channel and time codes are by no means mutually exclusive. Some systems might utilize rate coding, whereas others operate directly on temporal patterns and timings of spikes. A commonly held opinion is that nervous systems make use of both types of coding, with temporal codes being deployed in some sensory peripheries, while rate codes prevail in central stations. Channel activations and spike timing relations can also be combined to form composite, joint multimodal representations (the sides of the triangle in Figure 3A). Auditory examples of such joint codes are Licklider’s duplex model of pitch (Licklider, 1951), average localized synchronized firing rate (ALSR) profiles (Young and Sachs, 1979) and interval-place codes (Voigt et al., 1982) that combine temporal pattern and across channel synchronies with channel-based cochlear place information.

### 5.2 Channel Codes

In the vast majority of the neuroscience literature the conventional, default neural coding assumption is channel coding. Most often when neural coding is not explicitly discussed. The simplest channel-based codes are “doorbell codes” (also called “dedicated lines,” “labeled lines,” and “local codes”) in which selective, narrowly-tuned neurons fire only in response to some specific stimulus condition, such as the detection of a specific pheromone molecule. For the most part, neurons that respond differentially and monotonically to only one feature are rare in the CNS, such that, arguably, if combinations of attributes in multiple objects need to be represented, then either elaborate disambiguation mechanisms or some means of multiplexing the various neural response components is required.

In rate-channel codes, firing rate profiles of ensembles of neurons that are tuned with respect to some stimulus characteristic can convey stimulus information, such as through spatial patterns of excitation amongst sensory receptors. Rate codes assume some temporal integration (spike counting) time for individual neurons, typically on the order of tens of milliseconds or more. Because maximum driven firing rates of cortical pyramidal cells are relatively low, typically well less than 40 spikes/s, within a 50 ms spike counting window many neurons fire only up to 2–3 spikes. If sensory patterns can be discriminated in a matter of a few tens of milliseconds, then rate coding is not a viable means of representation (VanRullen et al., 2005). In order to encode more than a few rate-based distinctions within neurocomputationally relevant time windows, rate-based codes must pool spike counts from many neurons. That limitation notwithstanding, ensemble and population-level codes do potentially permit coding via the mass statistics of large numbers of neuronal responses, provided that their responses can be aggregated together and read out rapidly (MacLean and Hatsopoulos, 2019). Dense population codes rely on responses of large fractions of neuronal populations, whereas sparse population codes rely on small numbers of responding neurons. The fraction of responding neurons in a population, i.e., the extent of elevated neuronal activity, can also serve as a rate-like coding variable.

### 5.3 Temporal Pattern Codes

The simplest temporal pattern code is an interspike interval code in which information is encoded in time durations between two spikes. Interspike interval codes can be found in any sensory system in which there is time-locking of spikes to stimuli. Strong examples can be found in mechanoreception (flutter-vibration frequency, Werner and Mountcastle), audition (periodicity pitch, Meddis and Hewitt, 1991; Cariani and Delgutte, 1996a,b), and spatial vision. In visual thalamus, neurons robustly phase-lock to moving gratings to produce interspike intervals related to the temporal modulation frequency of luminance variations (Cariani, 2001b). Interspike interval differences can also exist in sensory systems, such as color vision (Kozak and Reitboeck, 1974), where characteristic, wavelength-specific interval patterns may be generated by differences in receptor response latencies and not through time-locked spiking.

**FIGURE 4:**
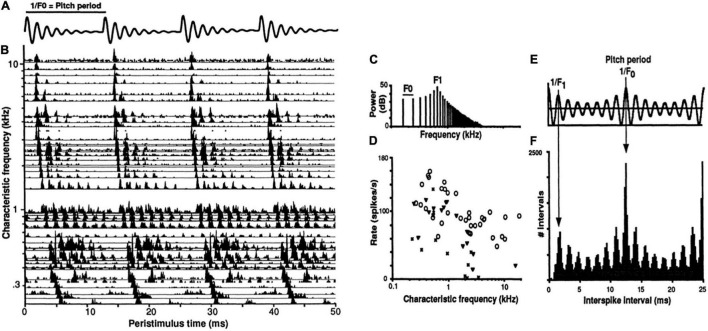
Temporal coding of pitch and timbre (vowel quality) in the auditory nerve. **(A)** Synthetic single formant vowel stimulus waveform (F0: 80 Hz, F1: 640 Hz, 60 dB SPL, 100 repetitions). **(B)** Post-stimulus time spike histograms of 42 auditory nerve fibers arranged by their characteristic frequencies (CFs). **(C)** Stimulus power spectrum. **(D)** Rate-place profile. **(E)** Stimulus autocorrelation function (ACF). **(F)** Population-interval distribution (PID) histogram of all-order interspike intervals of the whole ensemble. Delay intervals associated with major PID peaks closely correspond to the period of the perceived voice pitch (fundamental period 1/F0 = 12.5 ms). The pattern of minor peaks (0–5 ms) robustly encodes vowel formant structure and perceived aspects of timbre related to spectral shape. The PID provides a general purpose, neural representation of the stimulus autocorrelation function. The systematic first spike latency shifts in B are due to cochlear delays. From Cariani (1999).

Temporal coding of periodicity pitch (a.k.a. musical pitch, low pitch, F0-pitch) is a strong example of a temporal pattern code at the level of the auditory nerve (Cariani and Delgutte, 1996a,b; Cariani, 1999, 2019). Spike timings of auditory nerve fibers in response to a synthetic vowel are shown in Figure 4. A neural representation of pitch based on the population-wide distribution of all-order interspike intervals (time durations between both consecutive and non-consecutive spikes) predicts, with very few exceptions, all major monaural periodicity pitch perception phenomena below the 4 kHz limit of phase locking (Meddis and Hewitt, 1991; Meddis and O’Mard, 1997; Cariani, 1999): pure tones, harmonic and inharmonic complex tones with and without energy at the fundamental, repeating noise, musical dyads and triads, and spectral edge pitches. The F0-pitch that is perceived is accurately predicted (<1% error) by the predominance in the auditory nerve of interspike intervals corresponding to the same frequency and its subharmonics.

The temporal code for periodicity pitch at the level of the auditory nerve thus appears to be a population representation that relies entirely on the mass statistics of temporal spiking patterns and not at all on channel identities, i.e., it does not matter which neuron produced which spike train. One can discard all cochlear place information with essentially no functional consequence for the encoding of pitches below 4 kHz (cochlear place information is likely needed for pure tone frequencies above this limit).

Distributions of all-order intervals are equivalent to autocorrelation functions of spike trains. By virtue of phase-locking, each population-interval distribution also closely resembles the autocorrelation function of the acoustic stimulus (Figure 4E), enabling it to serve as precise and robust temporal representations of both the stimulus autocorrelation function and its power spectrum. Whenever stimulus-locked spiking exists, temporal pattern codes are well suited to provide autocorrelation-like representations of stimulus periodicities and low-frequency power spectra. Such representations can also encode overall stimulus intensity (loudness) using the ratio of stimulus-driven, synchronized spiking to uncorrelated, spontaneous activity (Cariani, 1999).

Temporal codes need not be synchronous across neural populations. In the auditory nerve example above (Figure 4B), if the spikes were summed together to form a population-post-stimulus time histogram, cochlear delays would smear out the fine timing patterns that exist from 200 Hz to ∼4 kHz such that the whole population response would only show timing information up to ∼200 Hz. Thus an absence of synchronous temporal patterning at the population level does not rule out higher frequency or asynchronous temporally patterned activity at lower, single neuron and ensemble levels.

Codes based on complex temporal patterns in the form of spike interval sequences, such as spike triplets (Strehler and Lestienne, 1986; Lestienne, 1996, 1999) and longer interval sequences (Emmers, 1981), are also possible. Spike timing, even at the cortical level, can be quite precise, in the sub-millisecond to millisecond range (Lestienne, 2001; Shmiel et al., 2005, 2006).

### 5.4 Time-of-Arrival Codes

**FIGURE 5:**
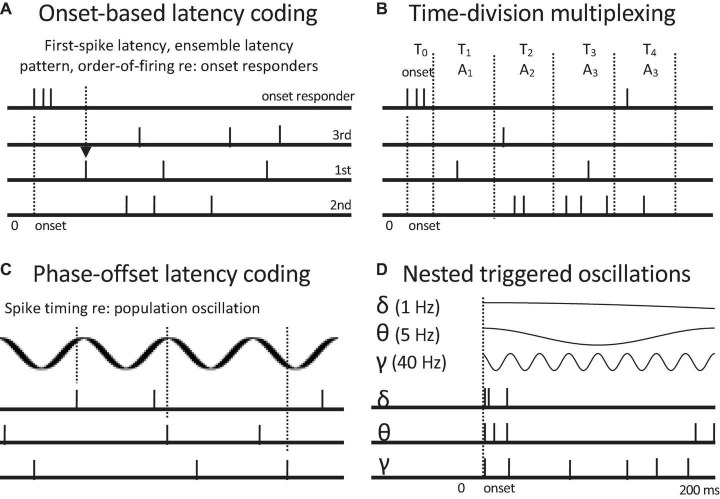
Neural codes based on spike latencies relative to onset responses and oscillations. Onset responses of whole populations or subpopulations of onset responders can serve as a reference time. **(A)** Onset-based latency coding. First-spike latency, order of firing, or post-onset ensemble volley pattern can encode profiles of attributes such as relative intensity, by marking specific channels. **(B)** Time division multiplexing. Different time post-onset time windows encode different event attributes, here encoded by different response channels or combinations of them. **(C)** Phase-offset latency coding. Spike timings at different oscillatory phases encode presence of different attributes. Alternately successive oscillatory cycles can temporally discretize population rate responses to convert to rate-sequence codes. **(D)** Nested oscillations with common onsets (phase resets). Three common oscillatory frequencies are shown. Nested oscillations can serve as coding frameworks for onset-based latency codes, time-division multiplexing, and phase-offset coding. They can also selectively activate different subpopulations. They potentially provide a common temporal framework for hierarchical temporal grouping of events such as the recognition of spoken words, phrases, and sentences. Oscillation-based codes can convey multiple attribute distinctions.

Time-of-arrival or spike latency codes are based on the relative timings of spikes across different channels or to a pattern of spike latencies following some specified reference time (Nádasdy, 2000). Whereas temporal pattern codes are indifferent to the precise times of occurrence of spike patterns, time-of-arrival codes depend entirely relative spike timing (Figure 5). Cross-channel latency codes use differences in spike latencies across channels to encode attributes such as sound direction. For example, those neurons in auditory cortex with the earliest first spike latencies provide the best information for sound location (Stecker and Middlebrooks, 2003). Temporal reference points can consist of spike timings in other (sets of) channels or spike latencies relative to onset responses or bursting patterns. Onset-based codes can be based on first-spike latencies (Figure 5A), complex spike latency patterns, or oscillatory phase (Figure 5C). Spike latency statistics, such as absolute latency, temporal dispersion, and degree of synchronization within a population, can convey information related to the intensity of a stimulus. The ordering of spike latencies permits ordinal, channel-sequence codes. Likewise, synchrony-based codes can be regarded as spike Latency codes can be regarded as “synchrony-based” under the senses of synchrony that involve specific relative delays between spikes and that involve spike timing relative to a temporal framework.

#### 5.4.1 Cross-Channel Relative Time-of-Arrival

Whenever there exists stimulus-locked spiking, spike latency codes are well suited to produce temporal cross-correlation-like representations of stimulus times-of-arrival at different sensory surfaces located at different places on the body (Cariani, 2001b).

A textbook example of spike relative latency coding involves sound localization in the horizontal plane. Sounds arriving at the two ears from different directions cause interaural time differences of up to hundreds of microseconds. By virtue of phase-locking, the interaural time differences produce spike timing differences between corresponding auditory nerve fibers that innervate right and left cochleas. Direction can then be inferred from an array of different delay paths that innervate interneural spike timing delays via neural coincidence detectors in the auditory brainstem that compute binaural cross-correlation functions (Cariani, 2011). Although spike jitters in single mammalian auditory nerve fibers are on the order of ∼100–200 μs, humans can discriminate interaural delays differing by ∼10–20 μs that correspond to differences of ∼1–2 degrees of azimuth. By comparison, best human pitch perception is on the order of 0.1% for 1 kHz pure tones, which corresponds to a period difference of 10 μs. Neural temporal correlation mechanisms for echolocation in bats and cetaceans and electroreception in weakly-electric fish are much more precise even than this.

#### 5.4.2 Time-of-Arrival Relative to a Reference

Comparative spike latencies across channels (latency-place codes) can also encode differences in response times of different receptors and tuned elements in their associated neural pathways. Population-wide distributions of spike arrival times relative to some reference time, such as an onset volley of spikes associated with stimulus onset and/or the beginning of an oscillatory period, can convey information about the relative activation of different classes of receptors. In this manner, relative ratios of class activations can potentially be computed from the temporal distribution of spike times without the necessity of keeping track of which neurons produced which particular spike latencies.

#### 5.4.3 Absolute First-Spike Latencies

Absolute response latencies relative to some reference time (which can be an early population response) can serve as indicators of stimulus intensity in different sensory channels (the more intense the stimulus, the shorter the response latency). Shorter first spike latencies are often correlated with higher firing rates, making the two codes sometimes difficult to disambiguate (Stecker and Middlebrooks, 2003). Relative average latencies across populations that are differentially sensitive to different aspects of the stimulus can indicate the relative ratios of those aspects. Temporal dispersion of first spike responses (variances of first-spike times, i.e., how temporally compact is the response) following the onset of a stimulus transient can serve as a measure of stimulus intensity.

#### 5.4.4 Complex Latency Patterns

**FIGURE 6:**
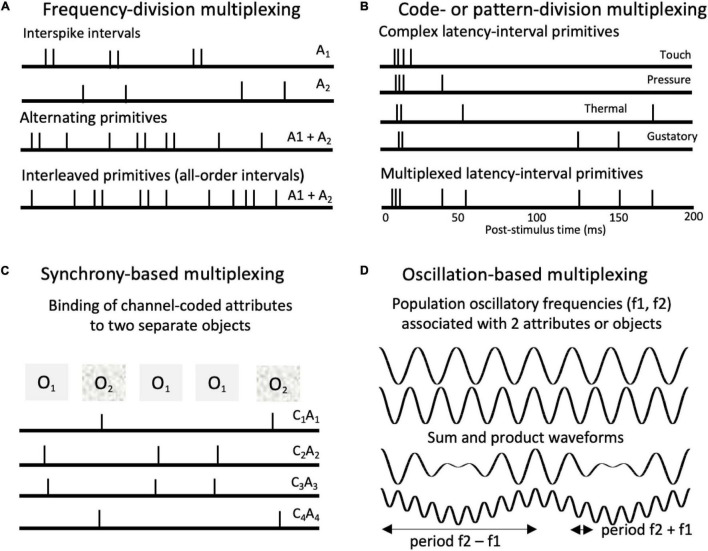
Types of multiplexing of pulse-coded signals. **(A)** Frequency-division multiplexing in which different attributes (A1, A2) are represented by different spiking periodicities (interspike intervals). Intervals associated with the different attributes can either alternate or interleave within spike trains. **(B)** Code- or pattern-division multiplexing. Spiking patterns associated with different cutaneous and gustatory tongue sensations (Emmers, 1969, 1981). The proposed codes involve characteristic latency-interval patterns relative to onset bursts. Such codes could be multiplexed in single neurons, ensembles, and populations. **(C)** Synchrony-based multiplexing. Synchronized spikes can support binding of attribute combinations associated with separate objects. Here spike synchronies across channels bind together channels (C_1_ – C_3_) that code for specific attributes (A_1_ – A_3_) associated with different objects (O_1_, O_2_). **(D)** Oscillation-based multiplexing. Top waveforms depict two population oscillations (f1 = 4 Hz, F2 = 5 Hz). Summing the waveforms produces an additional beat periodicity at (f2 – f1 = 1 Hz). Multiplying them produces two beat periodicities or sidebands (f2 – f1 = 1 Hz; f1 + f2 = 9 Hz), characteristic of the cross correlation of the two oscillations. Multiplicative combinations of frequencies create new frequencies.

Complex, spike latency spike patterns are also possible (Nádasdy, 2000). The patterns shown in Figure 6B were typical spiking responses of individual neurons to different modalities of stimulation on the tongue (Emmers, 1970). Emmers also reported finding evidence for a thalamic spike interval sequence code for pain in which multiple sequences were interleaved within the same spike trains (Emmers, 1981). These codes involved interval sequences with characteristic latencies that followed an onset burst, which appeared to serve as a temporal reference for the subsequent pattern (Middlebrooks et al., 1994, 1998).

Evidence for temporal pattern coding of taste qualities has been found in the gustatory pathway along with other labeled line, channel-pattern, and coarse temporal population rate codes (Hallock and Di Lorenzo, 2006; Ohla et al., 2019). The temporal codes are thought to be functional because corresponding temporal patterns of electrical stimulation produce characteristic orofacial behavioral signs of different taste classes (Di Lorenzo et al., 2009).

Yet another possibility would be temporal volley patterns that spanned different neurons in an ensemble or population, as might be produced in a synfire chain (Nádasdy, 2000). Although synfire chains are usually considered in terms of sequences of specific channel activations, each chain also produces characteristic temporal volley patterns that correspond to delay-coincidence paths. These volley patterns might also serve as complex temporal pattern and latency pattern codes.

#### 5.4.5 Oscillatory Phase-Offset Codes

Oscillatory phase-offset codes (Figure 5C) utilize the timings of spikes relative to a population oscillatory response to encode different types of information (Hopfield, 1995). These codes have mainly been studied in connection with olfaction and memory.

A host of oscillations related to sniffing cycles, attentional odor sampling, exist in the olfactory system (Buonviso et al., 2009; Kay et al., 2009). Phase-offset or onset-latency relative to sniffing cycles appears to provide a concentration-invariant code for odor identity (Schaefer and Margrie, 2012). Whether population-wide oscillations, as observed in local field potentials, are obligatory or facilitative has been a question. Abolition of some oscillatory behavior, such as abolition of theta rhythms by picrotoxin injection into the locust mushroom body, degrades fine, but not coarse, odor discrimination (Stopfer et al., 1997; Kauer, 1998; Lestienne, 1999). Likewise, manipulation of sniffing cycles and their mechanosensory correlates, also appears to degrade fine, but not coarse, odor discrimination.

Oscillatory phase-offset codes could operate in several ways. First, they could function as frameworks for time-division multiplexing in which different attributes, olfactory dimensions or perceptual features associated with a given place, could be encoded in successive gamma cycles. The gamma cycles would be nested within slower rhythms, within each sniffing cycle in olfaction and within theta cycles in hippocampus. Second, gamma cycles could discretize temporal rate responses, converting coarse temporal firing rate patterns into channel-based rate-sequence codes. Third, the codes themselves might be relative latency codes that are independent of the oscillations, but that the oscillations might improve temporal coding fidelity by reducing membrane noise, leading to lower spike timing jitter (Schaefer et al., 2006). This might explain why smell is only impaired but not abolished when olfactory oscillations are eliminated.

Oscillations and oscillatory phase-offset codes have been prominent in hippocampal memory research for several decades now (Skaggs et al., 1996) and have served as canonical examples of how neural codes might depend directly on population oscillations. A large literature has developed around phase-offsets in nested theta-gamma oscillations (Lisman, 2005; Dragoi and Buzsáki, 2006; Nádasdy, 2010; Buzsáki and Watson, 2012; Lisman and Jensen, 2013; Sanders et al., 2019).

Beginning in the 1970’s, hippocampal “place cells” were discovered that respond when rats enter a particular maze location, such that specific sequences of place cells fire when a particular path through the maze is traversed (Pavlides and Winson, 1989). In the late 1980’s it was discovered that the hippocampus produces these firing sequences during sleep stages (Pavlides and Winson, 1989), when short-term memory traces are consolidated into long-term memory stores. It was subsequently discovered that the hippocampus produces sped up, sequences of place cell firings during slow wave sleep (Lee and Wilson, 2002) in bursts of activity called sharp-wave ripples. The process has been labeled “hippocampal replay” (Findlay et al., 2020). A 20-fold time-compression has been recently observed in waking humans (Buch et al., 2021). “Time cells” that fire at times corresponding to when rats traversed different maze locations were discovered in hippocampus, cerebral cortex, and striatum (Eichenbaum, 2014). Time cells span a wide range of timescales (Howard and Eichenbaum, 2013) and the same hippocampal population can multiplex timing information on multiple timescales (Mau et al., 2018). In addition to pure place and time cells, units that respond to both place and time are found.

These findings beg the question of how all of the attributes related to navigation, are represented (neurally coded) and stored in memory (Howard et al., 2014; Eichenbaum, 2017; Lisman et al., 2017), presumably in one common representational form (Howard et al., 2014). Representations based on sequences of places, which could utilize channel order-of-firing codes and internally generated sequences (Buzsáki, 2013), would be possible. Alternately, representations that preserve metrical timing, such as channel-latency and temporal pattern codes, could also incorporate temporal context (Eichenbaum, 2017) and whole timelines of events. From conditioning studies, it has long been appreciated that brains have general mechanisms for reward prediction that assemble coherent timelines of unrewarded and rewarded events, even if only pairwise fragments of event sequences are presented (Miller and Barnet, 1993). Constructed timelines then can support prediction not only *that* a reward will occur, but also *when* in future time (Figure 2E). Hippocampal time compression appears to be roughly scale-invariant (Liu et al., 2019) enabling memories of past experiences to anticipate time courses of events so as to usefully guide prospective behavior (Tiganj et al., 2019).

Although great strides are being made in understanding the roles of place and time in navigation-related tasks, the problem of the neural coding of place itself is still unsolved. As Howard Eichenbaum remarked,

“The previously described studies provide compelling evidence that identifies the networks of neurons that encode memories and shows the specificity of particular sets of neurons that participate in an engram. However, these studies tell us nothing about the specific information encoded by the activated cells. They tell us nothing about the features of the learning events that are encoded by particular neurons or about the temporal patterns of activity in neurons and net- works that embody the information represented within the engram. They leave open the key question, what is the “memory code”?” (Eichenbaum, 2016).

### 5.5 Firing Order Codes

Ordinal, firing order codes rely on relative spike latencies of different channels to rank-order spikes (Figure 5A) (Thorpe, 1990; Nádasdy, 2000). Although both codes rely on spike latencies, firing order codes are grounded in ordinal time, whereas spike latency codes operate in metrical time (Figure 2). Whereas absolute latency and latency-pattern codes need not entail channel-coding, ordinal codes require the retention of channel-identities.

Spike ordering may require an onset event, such as an onset-triggered wave of inhibition, to provide a temporal reset or it can also be achieved by ordering spikes that occur in temporal clusters.

In contrast to rate codes, firing order codes enable extremely fast read-outs based on the earliest spiking responses that is needed to account for the rapidity of auditory, visual, and somatosensory discriminations, classifications, and recognitions (Gautrais and Thorpe, 1998; VanRullen et al., 2005). They work well in situations where only one spike is elicited per stimulus presentation (Van Rullen and Thorpe, 2001), where a spike rate is, strictly speaking, not even well-defined. Such codes have been proposed for vision and olfaction.

Such codes produce combinatorically-large numbers of firing order patterns (McCulloch, 1969) that can be discriminated by known neural mechanisms (Thorpe et al., 2001), and the ordinal sequences, like many percepts, are highly invariant with respect to time-warping transformations.

### 5.6 Multiplexed Coding Schemes

Multiplexing is the concurrent transmission of multiple independent signals over the same transmission line or channel (Figure 6). Multiplexing allows systems to gracefully handle high-dimensional representations. In neural contexts, multiplexing is most often conceived on the single neuron level, where a given spike train can convey multiple types of information over a single axonal transmission channel.

Perhaps the simplest examples involve temporal pattern interspike interval codes in which multiple types of information are carried via different interval periods (Figure 6A), that either alternate (Chung et al., 1970) or can also be interleaved (Emmers, 1981). Patterns related to multiple attributes of auditory events, such as loudness, pitch, timbre as well as rhythm are all present in spike trains of single auditory nerve fibers as well as in their mass statistics at the population level (§ 5.3, Figure 4). Multiplexed control of muscle extension and force has been found in invertebrate motor systems (Bittner, 1968). Multiplexing in axonal branches is also possible (Raymond and Lettvin, 1978; Waxman, 1978; Cariani, 1995a).

The mass statistics of temporal spike patterns can carry multidimensional information, as the example of the population-interval representation in the auditory nerve demonstrates (Figure 4). Multiplexing can also exist at the level of neuronal ensembles and populations, when the same sets of channels participate in concurrent representation of multiple objects. This can be realized by grouping spikes by different specific times (Figure 5B), oscillatory phases (Figures 5C, 6D), temporal patternings (e.g., burst patterns), or interneuronal synchronies (Figure 6C).

Multiplexing of signals can also exist at sub-neuronal and single neuron levels. To the extent that neuronal spike initiation and propagation through axonal branches is unitary, all inputs are summed together, and the resultant spike trains inherently reflect mixtures of signals. However, if there exist multiple independent sets of synaptic inputs capable of initiating spikes, multiple coincidence detection processes in (active) dendritic trees, or selective conduction failures in axonal branches (Waxman, 1978; Cariani, 1995a), then individual spikes in the same spike train may be parts of different larger spike patterns that are interleaved.

A general advantage of pure temporal codes over channel codes is that they can convey information without the necessity of retaining specific channel identities through specific transmission paths and connection weights. Population codes based on the mass statistics of temporal patterns of spikes liberate neural signals from specific transmission lines, making them resistant to disruption. In the population-interval coding example (§ 5.3, Figure 4), all channel information can be discarded without significant loss of function.

More complex temporal pattern and spike latency codes are possible in which multiple attributes can be represented in the same spike train. Combined with appropriate neural processing architectures, temporal codes permit multiplexing of signals at the single neuron level, such that multiple pulse-coded attributes can be conveyed in the same spike train. In contrast, rate-channel codes and rate-integrating neurons do not enable multiplexing at the single neuron level, because of the inherently scalar, one-dimensional nature of spike rates.

Multiplexing of information permits a given neuronal element to convey multidimensional information and allows one element to contribute to the representation of multiple perceptual objects, provided there is a means of associating signals (binding them) with the particular objects. Because there can be multiple signals transmitted through the same neural paths, multiplexing enables much more flexible communication than “switchboard” style networks (John, 1972). Because neural assemblies can pass through irrelevant signals and selectively respond to relevant ones, broadcast-based neural integrations and co-ordinations are also enabled.

#### 5.6.1 Time- and Frequency-Division Multiplexing

In terms of neural pulse code schemes, three strategies for multiplexing signals are time-division, frequency-division, and code-division multiplexing (Figure 6). Depending on how their neural signals are interpreted, oscillation-based coding schemes (Figure 6D) can fall under either frequency-division or time-division categories. In order not to conflate the two distinct senses of “frequency” as periodicity vs. temporal rate of discrete events (“spike frequency” codes based on firing rate) or prevalence in a sample (statistics), here we use the term strictly in its first sense, as in frequency-domain.

In time division multiplexing, time is divided into segments that can carry distinctions related to different attributes or attribute values. The segments can depend on the timing following a reference onset event or on a phase time position within a cycle. The cycle, such as a gamma cycle, may also be nested within a longer, theta cycle (Figure 5D), the same channels participate in the encoding of multiple objects albeit at different times. If objects are represented by patterns of channels (e.g., feature detectors) then common spike timing in subsets of channels can be used as a principle for grouping The lines remain labeled to signify the features they encode, while the time domain is used to signify which channels are grouped or separated. The temporal label can involve either timing relative to a reference wave or spike synchronization between channels.

Different types of information can also be multiplexed using multiple oscillatory frequencies of ensembles and populations to encode specific types of information. An example is “the spectral fingerprint hypothesis, which posits that different frequencies of oscillations underlie different cognitive operations” (Watrous et al., 2015).

In frequency-division multiplexing (Figure 6A), different signals utilize different frequency bands such that they can be mixed together in transmission channels and separated by receivers on the basis of their respective frequencies. In a pulse code, inter-pulse intervals can directly encode different frequencies. The population-interval representation of low frequency sounds discussed above (Figure 4) is a frequency-multiplexed system in that interspike intervals associated with different stimulus periodicities are concurrently conveyed by the same overlapping sets of auditory nerve fibers. In the visual system of the frog, different multiplexed intervals also can convey information about different aspects of the visual scene (Chung et al., 1970; Wasserman, 1992).

Whether multiple intervals must be alternated or whether they can be interleaved depends on the nature of the receivers that interprets the pulse trains. If the receiving system resets after each incoming pulse (first order interspike intervals), then intervals must alternate to avoid destructive interference. If the receiving system can register all-order intervals, i.e., between consecutive and non-consecutive spikes in an autocorrelation-like analysis, then it will be impervious to interleaving of different intervals.

#### 5.6.2 Oscillation-Based Multiplexing

Modulation of spiking by population-level behavior, through subthreshold inputs from synapses, gap junctions, or emphatic effects, enables multiplexing of neural signals and controls via dual local- and population-level paths (Kopell and LeMasson, 1994). The modulatory, oscillation pattern is superimposed on driven behavior, be it rate- or temporally-coded. Neural oscillations can potentially support all three types of multiplexing in time-, frequency-, and temporal pattern domains. Oscillations divide and discretize time, such that each oscillatory period can become a separate time division for differentiating different attributes (Figure 5B) or discretizing temporal trajectories of values, such as those related to relative stimulus intensities (Shamir et al., 2009).

Packets of time-divided attributes or attribute-trajectories can be associated with each discrete event by virtue of their membership within a common oscillatory structure that is demarcated by its frequency and timing. If oscillatory frequencies encode attributes, then multiple concurrent oscillations can carry multiple attributes associated with events and objects. The multiple oscillations can be bound by temporal contiguity, common phases (e.g., common trigger, phase reset times). If faster oscillations are nested within slower ones and scaled (e.g., *n* gamma cycles/theta period), then harmonic relations can also be a potential basis for grouping. Finally, interacting oscillations can potentially produce complex temporal oscillatory patterns that can serve as representations of conjunctions of attributes. For example, two spike train signals can be multiplexed by convolving them, and demultiplexed by deconvolution operations (Longuet-Higgins, 1989). Analogous operations on oscillatory patterns are discussed further below.

#### 5.6.3 Code-Division Multiplexing

In code division multiplexing used in cell phone and internet networks, packets of encoded information are transmitted via dynamically changing paths though the networks. The packets include a header that includes information about signal identity (here, its ultimate destination and protocols for message handling) and a payload that carries the information content of the packet, which represents either analog signals or symbolic digital message content. Code division multiplexing through packet switching permits flexible routing of information through a network, freeing the sender from the necessity of specifying a specific transmission path.

Code-division multiplexing in the form of packet-based communications have been hypothesized to support cortical coding through a mixture of characteristic neural response timings and average firing rates (Luczak et al., 2015). In a neural pulse code implementation, headers and payloads can be signaled by characteristic patterns of pulses. Code-division multiplexing could be implemented via complex temporal pattern and spike latency codes. Here multiple attributes have sets of different associated complex pulse patterns that may be alternated or non-destructively interleaved. The mixtures of pulse patterns can then be broadcast widely and selectively received by neuronal assemblies tuned to respond to particular patterns or types of patterns. Multiple types of signals might then be asynchronously sent over each transmission line and demultiplexed by appropriate receivers (see discussion of transceivers and radio systems below).

### 5.7 A Latency-Pattern Coding Framework for Multiple Attributes

Eventually, neuroscience will need to come to grips with the problem of how the sets of attributes associated with events and objects are encoded, integrated, processed, and stored and retrieved both in short-term and long-term memory. The attributes include not only basic perceptual distinctions, but also cognitive recognitions, emotional contexts, cognitive imperatives (current goals and tasks), perceived affordances, and prospective actions.

For individual, isolated auditory events, basic perceptual distinctions include loudness, timing, duration, pitch, timbre, location, and their various aspects (sub-dimensions). For a musical melodic sequence, each note would have a set of associated attributes that would include the basic attributes, plus their temporal context, plus attributes of the sequence, such as melodic, timbral, and rhythmic/metrical sequence and interrelations. The sequence can also produce cognitive recognitions (a familiar melody), emotional responses, aesthetic and motivational effects, and prime subsequent behavior.

We take as a working hypothesis that all those attributes can be organized, and if relevant, retained in short- and long-term memory. Other kinds of information such as visual percepts and motor responses (e.g., if the musical sequence is danceable) should also be capable of being integrated into working representations and memory traces. This begs the question of how all this information might be organized into a coherent whole, which in turn suggests the hypothesis of a common coding framework that can handle all kinds of informational distinctions. What is needed is a coding framework that is open-ended, capable of adding new attributes, i.e., expandable and annotative. The codes need not be interpretable throughout the whole system. There could well exist local codes in sensory areas that are modality specific, such that their readout requires the neural populations that initially produced them. Binding of local codes could be achieved though common, multimodal rhythmic patterns on global levels (Geiser et al., 2014).

**FIGURE 7:**
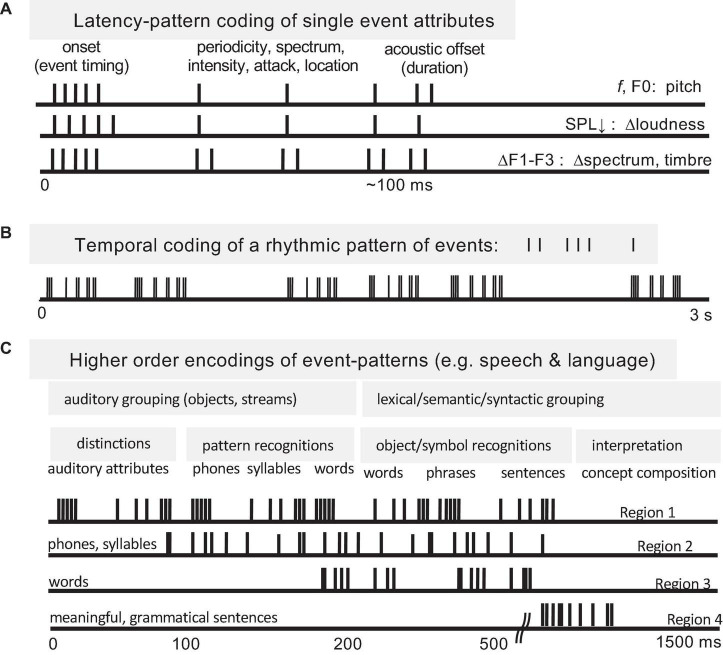
Proposed cortical latency-pattern coding framework for auditory events and speech reception. Onsets are neuronal bursts associated with transient acoustic contrasts. **(A)** Coding of perceptual attributes of auditory events by means of post-onset complex temporal spike patterns. **(B)** Coding of rhythmic patterns by temporal patternings of onset responses. **(C)** Coding of phones, syllables, words, and higher order syntactic and semantic relations in terms of onset-referenced latency-pattern codes in successive cortical regions. At each stage of the hierarchy, neural assemblies produce characteristic spike pattern markers associated with the recognition of acquired phonetic and linguistic distinctions.

A putative onset-based latency coding scheme for encoding attributes of auditory events and sequences of them is outlined in Figure 7. For individual auditory events (Figure 7A), such as an isolated musical note, a population onset response is followed by temporal patterns encoding different attributes such as intensity (loudness), periodicity (pitch), spectral shape (timbre_1_), attack (timbre_2_), and auditory location (direction). These temporal patterns are likely to only be partially visible at the population level, i.e., in evoked potentials. The end of the event is marked by an offset response with the interval between onset and offset responses encoding duration. The onset response encodes the timing of the event. A sequence of such events would be encoded by a time-series of pulse-coded packets, with the rhythmic pattern of the sequence being encoded by the temporal pattern of onsets (Figure 7B). The tonal structure of the melodic pattern would be encoded through interactions of the pitch portions of the individual events.

For speech (Figure 7C), we propose a ramifying annotative system in which incoming auditory neural representations are subjected to a series of pattern recognition operations carried out by neuronal assemblies. Upon recognizing a phonetic pattern, the assemblies emit a characteristic temporal pattern annotation. In this framework, top-down inputs can prime particular neuronal assemblies at any stage of processing. Later incoming inputs can also override earlier ones. Higher-level patterns of phonemic and syllabic tags are recognized as words by other neural assemblies, and lexical tags are subsequently emitted that activate spreading sets of concept nodes associated with the activations of still other neural assemblies. The concept nodes interact, resonate, and interfere to dynamically form an emergent, stabilized, compositional interpretation (meaning).

The inherent hierarchical structure of speech and language creates a natural test bed for analyzing and understanding spatio-temporal neural responses and relationships at multiple scales and levels. Starting at low level acoustic features, such as phones, (exemplars above), they progress to higher levels (e.g., Sahin et al., 2009; Singer, 2021): words (Marinković et al., 2003; Baker et al., 2011), sentences and semantic concepts (Mitchell et al., 2008; Chan et al., 2011). Evidence from fMRI, EEG, MEG, and eCoG, clearly support that both speech and language constructs are widely distributed over many cortical regions. They are dynamically and redundantly evident in time and space across the cerebral cortex (Blumstein and Amso, 2013; Ding et al., 2016; Hamilton et al., 2021; Stephen et al., 2021).

## 6. Experimental and Methodological Precepts

Through our experiences in neuroscience and engineering, we offer some suggestions to researchers seeking to understand how brains work. We recognize that change is often hard, but necessary.

(1) See the trees and the forest. If possible, observe the system at multiple levels. In order to observe the presence of precise temporal spike patternings, recording of spike trains from single and multiple units is essential, which necessitates invasive methods (ECoG, animal single unit studies) that drastically limit the numbers and types of experiments that can be done. Nevertheless, precise spiking patterns that have been observed in such experiments can serve as existence proofs for the possibility of temporal pulse codes, not only in sensory peripheries but in central stations as well.
(2) Try not to rule potential codes out of hand. One good example, if it can be reliably replicated, constitutes an existence proof. It is difficult to experimentally rule out many of the pulse coding schemes proposed above, especially if they are asynchronous, local temporal codes, i.e., not based on spiking that is synchronous at the population level. For the most part neuronal oscillations have been observed via gross electrical potentials and magnetic fields that reflect the synchronized component of population activity. Whereas any temporal structure that is observed at the population level necessarily reflects temporally patterned activity at subpopulation, ensemble, and individual neural levels, an absence of temporal patterning at the population level does not rule out temporally patterned activity at lower levels.
(3) Use methods of characterizing neural systems that are not biased toward particular types of neural codes. Linear system identification methods commonly used at cortical levels to characterize spectro-temporal receptive fields (STRFs) would completely miss interspike interval information in the auditory nerve that is highly predictive of pitch perception (§ 5.3, Figure 4).
(4) If you do not look, you will not see. The vast majority of single-unit studies do not look for embedded complex and interleaved, spike temporal patterns or cross-channel volley patterns because these are often not obvious in spike raster plots or post-stimulus time histograms. Such patterns have been found when investigators have looked for them with appropriate methods (Abeles et al., 1993; Abeles and Gat, 2001; Lestienne, 2001).
(5) If you filter out potentially relevant information, you can miss important things. Low-pass filtering of raw data in the belief that there is no observable or relevant structure there in higher frequencies is a pervasive problem. Electrical gross potentials and magnetic signals are often low-pass filtered at 200 Hz or below, eliminating any opportunity for observing temporal responses on finer scales than 10 ms, the Nyquist frequency being 100 Hz. As much as possible, we should maintain signal temporal resolution and integrity and examine the raw waveform data. Temporal resolution can often be improved simply by maintaining adequate sampling, avoiding under-sampling, by keeping sampling rates of the raw data at least 5–10 kHz. These measures would enable additional analysis and make possible the discovery of hitherto overlooked temporal fine structure.
(6) Although definitive answers to problems of neural coding may ultimately lie in spike train data, ongoing efforts a to improve the temporal resolution of non-invasive recording methods allow better visualization of fine time structure in population responses that can in turn suggest subsequent, higher resolution single and multi-unit experiments.
(7) Use as many alternative representations of stimuli and neural responses as possible. Each description may illuminate another aspect of the problem. Looking in the frequency-domain doesn’t preclude or replace time-domain descriptions and analyses. Combination, multi-domain descriptions are always possible.
(8) Keep things interpretable. Aside from making neural responses more difficult to interpret, excessively complex signal processing of neural data, especially using filtering operations, can generate frequency-domain artifacts that can be misinterpreted as oscillatory activity (Jones, 2016). Processing expediency and ingrained habits shouldn’t drive research. Be wary of data processing that is too complicated to be fully understood.
(9) Report precise values of response periodicities, i.e., in Hz with standard deviation error bars, and not just gross frequency ranges that span an octave or more.
(10) Use stimuli and tasks that are appropriate for what you seek to explain. Stimuli should be simple if the basic operation of the system is not yet well-understood. However, if one is seeking to understand how the system works in natural contexts, with all confounds and clues, then use natural stimuli. For speech there is a gamut of stimuli that range from clicks and tones to synthesized phones to isolated words and sentences to running, connected, conversational speech.
(11) If you don’t use at least some natural signals, you may miss whole aspects of neuronal response (Chan et al., 2014).
(12) Don’t assume that current models of neurons capture all functionally-relevant processes. Be wary of standard neural models. Our understanding of neurons is still not exhaustive. Textbook models often oversimplify. Some examples are: bidirectional gap junction synapses, axonal conduction blocks (Waxman, 1978), superexcitable phases of membrane recovery (Raymond and Lettvin, 1978), microtubule and molecularly mediated mechanisms, combined analog-digital signaling (Alle and Geiger, 2006, 2008), and inhibitory rebound effects on spike timing (Boudkkazi et al., 2007; Goel and Buonomano, 2016) and network synchrony (Rama et al., 2015).
(13) Be wary of predetermined anatomical structures and localized functions (function X occurs exclusively in structure Y or region Z). Don’t focus on only one prespecified region of interest (ROI). Don’t rule out involvement of connections with other, remote brain regions.
(14) Recognize that ideas are hard to change, on both individual psychological and social levels. The biggest impediments may the conceptual barriers inside our minds that need to be broken. Try not to reject out of hand ideas that go against conventional wisdom. Maintain a critical attitude toward all ideas, conventional and novel. Differentiate assumptions with weak evidentiary support from those with strong support. Provisionally trust, but continually test, established truths.
(15) Reject arguments based on authority rather than evidence. Nobody is infallible.
(16) Theorize like a physicist, experiment like a biologist, invent like an engineer.
(17) Try everything, test it, and keep what works (Thessalonians 5:21).

Our general precept is to try hard to maintain open minds about the nature of neural information processing, and whenever possible, to use methods that widen vision rather than narrow it with experimental and methodological blinders.

## 7. Neural Codes and Neural Network Architectures

Different types of neural codes require different types of neural signal processing architectures for their interpretation, i.e., readout that switches functional state or behavior. Within the coding typology of Abeles (1982a), basic types of neural processing architectures can be considered: (1) those that rely on dedicated lines (Abeles) and “switchboards” (John, 1972), such as traditional connectionist networks, (2) those that rely on “mass action,” i.e., statistical orders in neural populations (John, 1967b; Freeman, 1975), and (3) those that rely on delay-coincidence paths such as time-delay neural networks, synfire chains (Abeles, 1982a, 1990, 2003), polychronous networks (Izhikevich, 2006; Szatmary and Izhikevich, 2010), and neural timing nets (Cariani, 2001a, 2004, 2015).

By far, connectionist neural networks that are based entirely on channel-coding, have been most highly developed, culminating in contemporary many-layered, deep neural networks (DNNs). Channel codes are interpreted by architectures that can reliably distinguish channels with specific tunings or switch output channels that have consequent behavioral effects by virtue of specific channel connectivities. An example would be the mapping of movement direction neurons in motor cortex in which a direction-tuned neuron activates a combination of muscles that causes movement in a particular direction (Georgopoulos and Carpenter, 2015). Temporal pattern and spike timing codes are interpreted by architectures that have offsetting interneural delays that can either drive specific channels, as in time-delay neural networks and synfire chains, or produce temporally-structured output signals, as in neural timing nets. The population-interval coding of pitch (Figure 4) is an example of mass action in the form of population-wide interspike interval statistics. The kinds of complex, multiplexed spike latency pattern codes discussed above would presumably require decoding by networks that incorporate, one way or another, precise delays- and coincidence elements.

Sequential, purely connectionist networks discretize time into sequences of inputs and processing steps, operating on ordinal successions of time steps (machine cycles) rather than metrical time (Figure 2). Recurrent connectionist networks, while not explicitly introducing metrical time into their architectures, can, through recurrent loops, create discretized memories for sequences, as in the “nets with circles” of (McCulloch and Pitts, 1943) and the dynamic processing memory of recurrent networks of Elman (1990). Such networks can find structure in event sequences.

However, not all deep neural networks are based entirely on sequential channel-coding (Sejnowski, 2018). The broad, expansive category of DNNs can encompass subnetworks that incorporate time delays and coincidence elements as well as more traditional channel-based connectionist layers. In particular, convolution networks that adaptively configure their front-ends, and hence their feature primitives, can incorporate time- and frequency-domain representations and operations so as to deal with any temporal structure in their inputs that may be informative and robust. Such networks provide a partial solution to the unavoidable problem of choosing feature primitives that is many orders of magnitude more expedient than the time-consuming strategy in biological evolution of adaptively modifying or creating entirely new physical sensors (Cariani, 2012). Through adaptive modification, selection, and addition of feature primitives, highly complex, high dimensional classification problems under one feature set can be transformed into much simpler, lower dimensional problems in another.

As with the code types themselves, these types of functional architectures are not mutually exclusive, such that the nervous system could use fundamentally different types of neural information processing in different places or at different processing stages to realize different types of functions (Luczak et al., 2015).

We list in turn some general types of neural architectures that could handle the coding schemes discussed above: time-delay neural networks, oscillatory networks, synfire chains, wave interference networks and neural timing nets.

### 7.1 Time-Delay Neural Networks

Time-delay neural networks (TDNNs) consist of networks of interneural delays, coincidence detectors, and coincidence counters that interconvert patterns of spike timings into channel-activations and vice versa. Depending on their internal organization, TDNNs can act as pattern analyzers and pattern generators. By appropriate arrangements of delays, any incoming temporal pattern can be converted to a unique set of channel activations and any specific pattern of channel activations can be converted to a specific temporally-patterned output. Activated channels can be marked by differences in spike rates (coincidence counts) or the timing of output spikes. Time-delay networks can thus interconvert all three major types of codes discussed above (§5): channel, temporal pattern, and spike timing codes. Temporal patterns can also be transformed into ordinal firing sequences which can then be transformed to channel-coded outputs (Thorpe et al., 2001). Thus, all of the codes discussed above could be converted to channel- and rate-codes via TDNNs.

The first neural network models to successfully account for significant bodies of perceptual phenomena were the time-delay architectures of Jeffress (1948) and Licklider (1951). The Jeffress Model (Jeffress, 1948) aimed to explain how auditory localization could be achieved on the basis of interaural timing cues, whereas Licklider’s duplex place-time architecture (Licklider, 1951) provided a theory of the auditory representation of periodicity pitch and power spectrum. Whereas the Jeffress architecture computed a binaural cross-correlation function, Licklider’s duplex computed spike autocorrelations within a set of spectral, cochlear place channels.

The signal processing stages of Jeffress model entailed phase-locking of primary and secondary neurons in right and left auditory pathways, a set of brainstem conduction delay lines, a set of spiking binaural coincidence detectors, followed coincidence-counting stage (spike rate integrator). Through this architecture, the direction of a sound in the horizontal plane is inferred from a peak in the rate-channel profile of coincidence counts. The signal processing stages of the Licklider model similarly relied on phase-locked spikes in each cochlear frequency channel, an array of chains of synaptic delays, and coincidence detectors, followed by coincidence counters. Licklider’s later “triplex” (Licklider, 1959) and Cherry’s “two ears” (Cherry, 1961) architectures integrated monaural temporal autocorrelations and rate-place power spectra with binaural cross-correlations.

In the years following these early neural time-delay networks, analogous networks were proposed for cerebellar timing functions (Braitenberg, 1961, 1967, 2000) and visual pattern and motion detection (Reichardt, 1961). Networks with adaptively modified conduction delays were also proposed (MacKay, 1962).

Neural delays can come from a variety of processes, including synaptic, conduction, integrate-and-fire dynamics, and rebound from inhibition. By appropriate arrangements of delays, essentially any temporal pattern of spikes can be offset by corresponding delays to produce temporal pattern detectors selective for that pattern (Torras, 1985; Gutig and Sompolinsky, 2006), thus effecting time-to-channel or time-to-rate transformations (Tank and Hopfield, 1987; Hopfield, 1996). Likewise, activation of an appropriately structured TDNN by one trigger can generate any temporal pattern, a basic capacity of brain function (Singer, 2019). Interneural delays can be adaptively modified by selectively increasing synaptic weights associated with particular delays. Effective connectivities between neurons could also potentially be modified by adaptively changing neural path delays, through alterations of axonal conduction velocities or timings of inhibition rebounds, that optimize particular rewarded coincidences.

### 7.2 Synfire Chains and Polychronous Networks

Synfire chains are general neural networks that consist of a set of delay paths and coincidence elements (Abeles, 1982a, 1990, 2003; Lestienne, 1999). The coincidence elements have short, sub-millisecond to milliseconds, spike integration times. As such, they are similar to time-delay networks, but without spike counters that read-out firing rates. They can incorporate both excitatory and inhibitory couplings. In feedforward synfire chains, volleys of spikes percolate through network delay paths to realize successive sets of temporal synaptic coincidences that produce output spikes that then propagate further through the network. Feedforward chains produce waves of volleys that can support onset-latency and firing order codes and whose interactions can be considered in terms of wave-interference networks. In recurrent, synfire cycles, activity can be self-sustaining, and therefore potentially capable of supporting reverberating short-term memory (Moradi, 2004).

Depending upon how their spiking outputs are interpreted by the rest of the system, synfire chains can potentially support different types of neural codes: channel coding (which particular neurons are activated), channel-sequence coding (firing order amongst neurons), latency and temporal pattern codes (temporal volley patterns of spikes), and combination channel-temporal codes (specific across-neuron “spatiotemporal” volley patterns). Potential drawbacks of synfire networks concern their robustness in the face of internal and external noise, but these may be surmountable if there are many such networks operating in parallel.

Compositionality and systematicity are fundamental capabilities of representational systems in perceptual, cognitive, mnemonic, and motor domains that are readily implementable through classical logics (Bienenstock and Geman, 1995; Touretsky, 1995). Following the resurgence of “connectionist” neural networks in the mid-1980’s under the banner of parallel distributed processing (PDP), a debate ensued about whether neural networks might be capable of realizing these mental functions. Early on it was proposed that binding operations based on neural synchrony might be adequate to support such functions (Ajjanagadde and Shastri, 1991). Parallel theoretical studies of synfire networks have also been undertaken (Abeles et al., 2004; Hayon et al., 2005).

Polychronous networks are “spiking networks that can polychromize, that is exhibit reproducible time-locked but not synchronous firing patterns with millisecond precision, as in synfire braids. The network consists of cortical spiking neurons with axonal conduction delays and spike-timing-dependent plasticity (STDP, §3.2)” (Izhikevich, 2006). This work emerged from large scale, 100k element, neuronal simulations (Izhikevich et al., 2004). Neuronal ensembles and populations in polychronous networks support multiple, concurrent synfire braids (waves) and therefore can multiplex signals encoded in the various synfire chains. Recurrent polychronous networks are assemblages of synfire cycles that can potentially support asynchronous spatiotemporal spike-timing patterns in reverberating, working memory (Szatmary and Izhikevich, 2010).

### 7.3 Wave Interference Networks

Spatiotemporal waves of spiking are observed in many sensory systems and as a rule, low frequency brain rhythms are not stationary but travel in waves from region to region (Nunez, 2000; Nunez and Srinivasan, 2006; Thorpe et al., 2007; Bhattacharya et al., 2022) and within structures such as the cerebellum (Braitenberg et al., 1997) and basal ganglia. As with onset events and stationary oscillations (Pouzzner, 2020), spatiotemporal waves can provide reference timings for both spike latency codes and ordinal, firing sequence codes. As with interactions between oscillatory frequencies and phases, interactions of waves can potentially be used for neurocomputational operations, such as informational organization and integration and the creation of new, emergent, more complex temporal patterns (§ 8).

Wave interference networks are neural networks that utilize the dynamics of interacting waves of spikes and population responses to encode and process information (Heinz, 2004, 2010; Izhikevich and Hoppensteadt, 2009; Kashchenko, 2015; Highland and Hart, 2016). Perhaps the earliest wave interference network was the “grassfire model” proposed by (Beurle, 1956). Wave interference networks can be regarded in terms of traveling wave oscillations that operate in “wave time” (Figure 2D). Wave interference networks potentially provide means of decoding latency-place codes as well as those based on oscillatory frequency or phase-offsets.

### 7.4 Oscillatory Networks

Cyclic, oscillatory behaviors are ubiquitous in biological systems. Early conceptions of brain functions imagined neural signals as miniscule vibrations, “vibratuncles” (Wade, 2005). Individual neurons can be modeled as non-linear oscillators with natural resonances and firing modes (Izhikevich, 2001). The repetitive motor actions that subserve most animal movement directly suggests pattern generation mechanisms consisting of systems of oscillatory elements (Gallistel, 1980). Consequently, networks of neural oscillators have been proposed both as general models for brain function (Greene, 1962; Amit, 1989; Hoppensteadt, 1997; Kashchenko, 2015) and for specific mental functions such as rhythm perception (Tal et al., 2017) and visual scene analysis (Baldi and Meir, 1990).

### 7.5 Neural Timing Nets

Whereas synfire chains and polychronous networks were devised from the bottom up, starting with the behavior of ensembles and populations of hypothetical neurons, neural timing nets were developed with specific perceptual functions in mind: problems related to auditory temporal coding of pitch and rhythm, perceptual invariance and equivalence, extraction of common pitch irrespective of timbre and vice versa, and separation of auditory objects of different fundamental frequencies (Cariani, 2001a, 2002, 2004).

Neural timing nets, like synfire chains, consist of arrays of interneural delays and coincidence detectors, albeit with delays arranged in a systematic fashion to facilitate interpretation and understanding. Timing nets take temporally-coded pulse trains as inputs and output other temporally-coded trains. The coincidence detectors multiply pulse inputs (AND operations). How the neural delays are realized biophysically (e.g., through axonal conduction, synaptic transmission, membrane recovery, and integrate-and-fire) does not affect the behavior of the networks. Operating entirely in the time domain, the networks compute auto-correlation, cross-correlation and convolution operations on pulse train inputs. An output set of spike trains is produced whose population-interval (summary autocorrelation) can serve as a representation of periodicity and timbre. Neural timing nets permit mixtures of temporal pulse patterns to be disambiguated, enabling demultiplexing of interleaved spike patterns.

Neural timing nets have been applied to problems of speaker separation by means of different fundamental voice pitches (F0s), with patterns of performance that resemble those of human listeners. They have been applied to the problem of rhythmic pattern induction, the grouping of repeating, arbitrary temporal patterns of musical events into a “groove” and the collateral expectancies that are produced.

**FIGURE 8:**
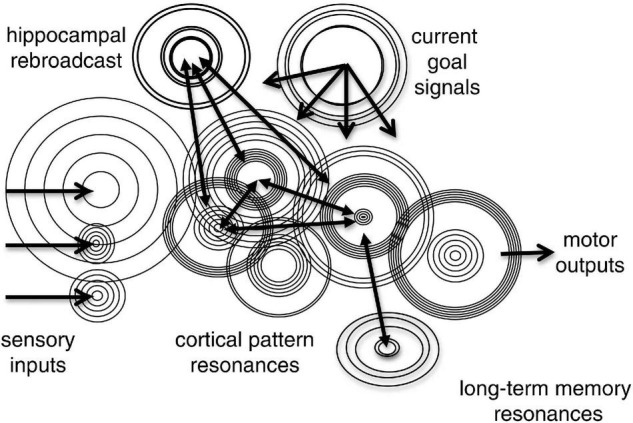
Pattern-resonance scheme for neural signal dynamics. Complex temporal patterns of spikes percolate through synfire delay-coincidence networks activating pattern resonances, forming characteristic spike interference patterns. Grouping is achieved through common spike sub-patterns at local levels and event-rhythm patterns at global levels. Spreading activation creates provisionally stable standing wave patterns in recurrent synfire-chain delay loops. Temporal patterns associated with current goals are injected into the network to amplify task-relevant patterns and suppress irrelevant signals and circuits. Temporal patterns in reverberating short-term memory activate similar patterns and sub-patterns in long-term memory traces that encode “tape-recorder” event-and-reward sequences that serve as anticipatory guides for prospective behavior (Cariani, 2017). Schematic from (Cariani, 2015).

As such, neural timing nets represent an analog, time-domain, relational, correlational approach to neural signal processing. By retaining temporal patterns of spikes in a reverberating memory and using them as matched filters for incoming signals, highly sensitive discriminations can be achieved. Such networks are “signal-centric” rather than “connection-centric” in that the computations involve signal interactions rather than structured, weighted connections between channels of feature detectors and association units (Figure 8). Virtually all of the action is, as in the auditory nerve example in § 4.3, in the temporal patterning of spikes.

Temporal codes and timing nets provide examples of how signals and signal processing need not be tied down to particular, identified neural channels, thus “liberating neural signals from wires” and enabling radio-like broadcast and selective reception strategies for neural communications, integrations, and co-ordinations.

Although conceived with auditory neural representations and Gestaltist grouping processes in mind, to the extent that central neurons are primarily coincidence elements and that central temporal codes exist, i.e., “temporal coding all the way up,” neural timing nets can be posited as the basis for alternative temporal theories of brain function (Cariani, 2015, 2017). These alternatives rely on principles of mass action, signal multiplexing, broadcast communication, active regeneration of signals in reverberant delay loops using complex temporal ensemble and population codes and dynamic, adaptive delay-computations effected in synfire chains.

This perspective bears similarities to Lashley’s ideas of non-local representations, mass action, and neural interference patterns (Lashley, 1998), John’s statistical temporal orders (John, 1972; Thatcher and John, 1977; John and Schwartz, 1978), Lorente’s and Kubie’s reverberating circuits (Kubie, 1930; Lorente de Nó and Fulton, 1949), Pribram’s holonomic orders (Pribram, 1971, 1991), and Longuet-Higgins’ holographic storage of time patterns (Longuet-Higgins, 1987a, 1989). To these ideas, we add the notion of a system based on local asynchronous temporal pattern codes stabilized through dynamically facilitated polychronous synfire cycles. Event- and object-related temporal representations from local regions would be bound together at global levels through common coarse temporal rhythmic patterns (cf. Geiser et al., 2014).

If everything can be kept in the time domain, encoded in spike temporal patternings and sequences, then “tape recorder” memory mechanisms are made possible in which temporal memory traces actively regenerated in reverberating circuits can be read out in faster-than-real time, as in hippocampal-cortical replay (Buch et al., 2021), to serve as anticipatory guides for effective action (Cariani, 2017). Temporal memory traces containing neural encodings of past events, actions taken, and reward outcomes can then predict future consequences of present courses of action based on past experience. A molecular mechanism for non-labile, long-term memory storage of temporal patterns is also conceivable if temporal spiking patterns can be mapped onto spatial patterns of side-chains in linear polymers. Disparate fragments of temporal sequences would be assembled into whole timelines through iterative interactions between traces with overlapping spike patterns. As with the genetic code, by keeping information in one temporal framework, many fundamental organizational problems of communication, coordination, and memory can be radically simplified.

## 8. Toward an Integrated Systems View of Brain Function

Despite decades of painstaking work that have produced a tremendous and rapidly growing body of specialized knowledge about the brain, we still lack integrated frameworks that compile and coalesce our functional knowledge of neurocomputational representations and operations into a coherent whole. We do not, by any means, claim to have discovered this holy grail, or even a sure path toward it. However, we can point to and suggest some new approaches for exploring this space with reference to some specific metaphors, models, and methodologies that we believe will be helpful.

These next sections outline the operations and characteristics of radio communication systems, transceivers, heterodyne receivers, reverberant circuits, and holography. It relates and contrasts these with comparable neural operations including temporal codes, multiplexing, phase-locking, oscillations, synchrony, memory, attention, anticipation, perception, and cognition.

### 8.1 Dynamic Transceiver Model Functions and Elements

The Dynamic Transceiver Model (DTM) is a conceptual mechanistic network architecture. It compares and contrasts the more elusive functions and elements in the brain with similar functions and elements of well-understood and tested broadcast transmission systems. The ubiquity and proven success of broadcast transmission systems such as cell phones, radio, and satellites, merit examination of DTM’s fundamental mechanisms, strengths and weaknesses. Despite obvious differences between these artificial and biological systems, at all scales of their operations, there are striking parallels that may provide insights that lead to better understanding the functions and capabilities of communications systems and brains.

Radio metaphors for the brain are not new though they typically have been applied to single components or operations within a communications system (Hoppensteadt and Izhikevich, 1998; Shanahan, 2008; Izhikevich and Hoppensteadt, 2009; Highland and Hart, 2016; Soman et al., 2018). The metaphors operate on several levels of structure and function, to compare radio components, circuits, and networks with neurons, ensembles, and populations and radio signals and signal processing operations with neural codes and computations. A key rationale for looking at the radio communications metaphor described here is that it comprises a complete end-to-end systems perspective, with integrated components.

In particular, it is hoped that this model may suggest rigorous experiments, especially for the roles of temporal coding, phase-locking, oscillations, synchronicity, temporal coupling, integration of time- and frequency-domain operations, memory access/retrieval, signal-to-noise ratio (SNR) enhancement, information integration, etc. Given the striking advances in neuroscience tools and computational processing, such studies are progressively feasible.

#### 8.1.1 Radio Analogy

**FIGURE 9:**
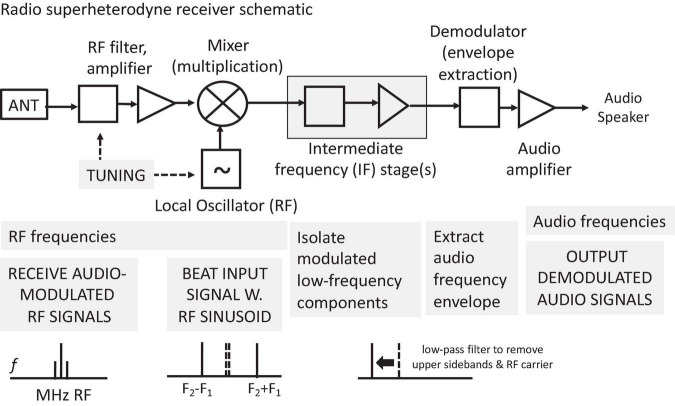
Schematic of a generic radio superheterodyne receiver.

A basic radio broadcast transmission system takes a given audio source signal from a microphone or recording, modulates its amplitude (AM), or frequency (FM), with or without phase modulation (PM), and combines it with a designated channel frequency carrier signal (Figure 9). The primary signal, the audio signal that contains the information of interest is combined with the carrier through modulation, i.e., a modification of the carrier’s amplitude (AM), frequency (FM), or phase (PM), to produce a composite modulation signal that contains both the audio source and the carrier. This composite modulation signal is then amplified, and wirelessly broadcast from a transmission tower. In the process of transmission, this modulation signal can be propagated, possibly through multiple amplification relays, before it is picked up by the antenna of a given radio receiver. The antenna is typically exposed to thousands of different signals. The radio receiver uses multiple tuning circuits to precisely select from all these signals, a designated carrier wave frequency with which it resonates. Matched filters and correlation receivers optimize SNR. A demodulator then takes the modulated signal wave and filters out the carrier signal and other unwanted signals from it. Finally, an amplifier then amplifies the original source signal for output through speakers or headphones.

Many radio receivers improve performance, accuracy, and reduce power requirements by also incorporating superheterodyne circuits that combine modulation signals with local oscillator frequencies to create higher sum (F2 + F1) and lower frequency difference (F2 − F1) sideband signals for subsequent processing. Typically the higher frequency sideband is low-pass filtered out, effectively downsampling and reducing the dimensionality of the modulation signal being transmitted. Multiple “intermediate filter” (IF) stages can be added to iteratively narrow bandwidth, thereby sharpening the tuning, and enhancing SNR performance. This process efficiently converts high frequency signals to lower frequency signals while maintaining informational integrity as well as reducing noise.

Transmitters and antennas can be designed for directional or omnidirectional operation. Devices that combine both transmitters and receivers for concurrent bidirectional communication (like telephones which enable users both to speak and listen at the same time, using separate carrier frequencies), are called “transceivers.”

#### 8.1.2 Characteristics of Radio and Neural Transceivers

Radio communication systems and neural systems demonstrably share many comparable functions at multiple levels. In addition to broad similarities in broadcasting, receiving, and transmitting signals, they share similar operations. These include the broad distribution of signals, modulating and demodulating them, suppressing noise, filtering, amplification, precision tuning, etc. Although the techniques clearly differ, examining parallels can be instructive in better understanding the signals and the mechanisms for manipulating them.

The signals of the two systems can be compared. Wireless radio waves are electromagnetic in nature, and can penetrate through many physical materials and obstructions. They span a frequency range of about 3 kHz–300 GHz. In wireless transmission systems, the radio waves travel close to the speed of light. By contrast, the electrical spikes propagated by mammalian nervous systems have velocities of ∼0.3–120 m/sec, with myelinated axons an order of magnitude faster than unmyelinated ones. For example, auditory nerve fibers show typical conduction velocities of ∼15 m/sec. Multistage neuronal paths and circuits incur delays at each synapse of roughly half a millisecond.

Our contemporary communications systems are largely wireless, whereas nervous systems are largely wired. A vast variety of radio communication systems includes RFID chips, radio and TV broadcasts, cell phones, GPS, wireless networking, WiFi, Internet, radar, satellite communications, etc. Although traditional landline phones use wires for signal transmission, newer units with wireless handsets, combine wired connections with short distance wireless radio transmissions between base units and handsets. Aside from local electrochemical influences and local field potentials, the nervous system of higher order animals, is densely wired, in series and in parallel, within an integrated network of networks. Interconnected cell populations and ensembles operate both locally and remotely, to serve specialized functions within the global network.

Temporal coupling of associated networks, especially via phase-locking, is widely observed across the brain and nervous system (Gupta and Bahmer, 2021). It is a critical mechanism for initiating and coordinating spatio-temporal coding, event detection, state changes, resetting voltage, and synchronization. In the context of radio systems, phase-locking is a fundamental operation, broadly used to detect, track, synchronize, and demodulate signals.

In contrast to radio systems, the brain and nervous system are highly adaptive and plastic. Neural circuits can progressively and autonomously produce more powerful signals as they are repeatedly activated. The circuits are also subject to degradation with disuse or damage. Although radio transmitters can broadcast with stronger or reduced power, and can be damaged, they do not change their broadcast power as a function of activation.

At multiple levels, neural systems dynamically change their characteristics and behaviors, including their connectivities and activations, as a function of their operations over time. Understanding the dynamics of neuronal networks, is key to understanding neural mechanisms and operations (Kopell et al., 2014).

The biological system, as a whole, can assume different modes e.g., awake, attentive, sleep, etc. Most neurally mediated autonomic functions, such as breathing control, digestive functions, etc., operate across all these modes. Although not bounded by it, this discussion of the DTM focuses on communications and behaviors of the system in the awake, attention-driven mode.

External sensory signals bombard the organism with an ongoing barrage of visual, auditory, etc. inputs. The organism is also bombarded by an ongoing barrage of internal physiological signals as well as by internally generated goals, thoughts, emotions, etc. By quelling most of this cacophony, the organism can focus and extract salient features from this environment for further processing (conscious and unconscious) and subsequent actions. Attentional mechanisms are responsible for directing an action-driven focus on relevant internal and external information. Analogous functions in radio communications systems, are served by transceivers, antennas, filters, lock-in amplifiers, tuning circuits, etc. that suppress noise, tune in and amplify signals of interest.

#### 8.1.3 Communications Operations in Radios and Brains

Communication system components typically consist of transducers, modulators/demodulators, transmitters, receivers, storage buffers, relays, amplifiers, automatic gain controllers, filters, mixers, tuning circuits, signal detectors, and antennas. Neural system analogs to each of these components are described below. Analogous operations are italicized.

In the nervous systems, sensory organs *detect* stimuli, *transduce* and *code* them into temporally patterned pulse trains of electrical spikes. In sensory systems, afferent pathways relay these spike sequences to more central processing sites, and efferent pathways relay spike sequences back from more central stations. Each station is a transceiver that can both *transmit* and propagate such signals afferently, up the pathway, and efferently, back down the pathway. Lateral paths within each station and local interneurons can provide cross-channel communication, which may influence subsequent transmissions. The spike train signals themselves may be *modulated* by underlying temporal or frequency carriers in the form of neuronal synchronies or oscillations.

Endogenous *oscillations* (e.g., alpha, beta, gamma, delta, and theta) are routinely observed, though there is great debate about their sources, mechanisms, roles, etc. (Siegel et al., 2009; Zoefel et al., 2018). Such oscillations can be coupled, and correlated with observable brain functions (Buzsáki and Wang, 2012). These oscillations are likely instrumental in serving different functions (e.g., amplifiers, carriers, modulators, etc.) at multiple levels. More speculatively, they may be related to or emergent from signal correlation and tuning operations, analogous to radio mixer local oscillators generating intermediate frequencies from interacting (e.g., mixing) with modulated signals (Figure 9). Brain heterodyne-like operations might be key to shifting, tuning, and matching networks with different operating characteristics across levels. Such a mechanism could efficiently and flexibly couple disparate reverberant networks both locally and remotely, temporally and spatially.

As previously described, heterodyning is a flexible mechanism for shifting (e.g., downsampling) and coupling signals operating over different frequencies, typically by multiplying a modulation signal with a higher frequency local oscillator (Figure 6D). This operation generates two sideband frequencies, one at a sum frequency (F1 + F2), and one at a difference frequency (F2 − F1). Typically the sum frequency sideband is filtered out, leaving a single sideband at the lower difference frequency (Figure 9).

Consider a hierarchical system, roughly analogous with a speech/language understanding system, characterized by a series of 6 lower level (higher frequency) to higher level (lower frequency) stages of integration; e.g., (1) sounds/phones, (2) phones/syllables, (3) syllables/words, (4) words/sentences, (5) sentences, (6) concepts/understanding. Suppose the signals from each stage in this hierarchy interact with the signals at the next higher level stage (e.g., a sequence of incoming phones gets integrated into syllables, syllables integrated into words, etc.). Hypothetically, each lower level signal might act as a local oscillator to correlate (e.g., multiply) their signal with those at a higher level. Suppose the modulated signals for the sounds/phones (lowest level) stage are characterized by a 45 Hz (gamma oscillation), and that successive levels of integration are characterized at approximately 22 Hz (beta), 12 Hz (alpha), 7 Hz (theta), 2.5 Hz (delta), and 1.5 Hz (low delta), respectively. If each stage is iteratively pairwise combined (e.g., mixed) with its next higher level stage, then the low frequency side band (beat) oscillations will be at 45, 23, 11, 4, and 1.5 Hz. This sideband oscillation sequence approximately recapitulates the standard sequence of endogenous oscillations itself, eventually downsampling by ∼30x.

A wide variety of inter-relationships of endogenous oscillations have been proposed; such as feed forward theta-gamma nesting to reflect hierarchical grouping of information for speech processing (Ghitza, 2020), bottom-up gamma combined with top-down beta (Noda et al., 2017) for predictive timing and error correction, theta-gamma codes for memory processes (Lisman and Jensen, 2013), and system level temporal dynamics for regulating attention and knowledge access (Klimesch, 2012).

In terms of the radio metaphor, neuronal cell ensembles create functional units that are in effect *broadcasting* spike train signals and their aggregated derivative features (e.g., firing rate temporal envelopes), throughout the network. Different channels constitute different *delay lines*. Temporally precise neuronal rebound processes also impose neural delays. Multiple neuronal pathways in cell ensembles provide robust redundancy though parallel distribution. A process where an input signal is modified through different network paths that are later synchronized and compared, can act as an error detection system. A given input signal processed through multiple parallel network paths can stimulate both fast responses as well as more extensively processed slower responses. The vast numbers of neurons, their dense interconnectivity, and plasticity create a dynamic self-organized infrastructure well–suited to support virtually any network functionally needed.

Neuronal elements may be excitatory or inhibitory and most neuronal circuits consist of both. The combination and interaction of these opposing influences determine the operations and behaviors of the neural system.

In multistage neuronal pathways, early stage neurons make synaptic connections both with lateral and later stage neurons at synaptic junctions. Trees of branching synaptic relays themselves can be regarded as *broadcast transmitters*. The post-synaptic stage can be regarded as a *receiver* of signals from the presynaptic stage. Signals of different strengths may or may not be adequate to be propagated by the next level. If the electrochemical characteristics of the two stages are conducive; that is, suprathreshold levels are obtained, the signal can continue to propagate to later stages for further *transmission*. When suprathreshold levels are not met, the post-synaptic stage may become more or less excitable, with subthreshold facilitation or inhibition that predisposes its responses to subsequent activations.

Neuronal junctures or synapses of all types, serve as relays, and are necessary in the configuration of multistage transmissions and channel circuits. At the synaptic junctures, the signal may be *amplified*, *attenuated*, or otherwise modified. Such modifications can effectively operate as *modulation* or *demodulation* processes. As in AM radio, a phase-locked envelope detector could serve as a simple half-wave (single side-band) *rectifier* to demodulate such signals.

In multichannel radio transmission systems, multiple data buffers may be incorporated for temporary data storage. Analogously, echoic (e.g., iconic visual and haptic) memory and working memory could operate as such temporary data *storage buffers*. Sensory memory buffers can range from less than a 1 s (iconic) to several seconds (haptic and echoic). Working memory, involving higher level executive functions, lasts longer, about 10–15 s.

In a radio system, a data controller directs traffic, selecting the number and type of radio channels to be utilized. Since the flow of incoming information can be irregular, the data controller manages the flow of buffered data through the radio channels to enable smooth transmission and reception. In neural systems, analogous informational gating and control mechanisms based on synchronies and oscillations could serve similar functions. Such a role has been proposed for cerebellar coordination of neuronal oscillation coherence in communications across multiple cortical structures (McAfee et al., 2021).

### 8.2 Content-Addressable Associative Networks

In radio broadcast systems, incoming signal information is automatically coded, transmitted, modulated/demodulated, and subsequently extracted. In the brain, incoming signal information is analogously automatically coded (e.g., through spike train sequences, phase-locking, spectral characteristics, etc.), transmitted, modified through multiple stages, and subsequently extracted.

Analogously, sensory organs transduce incoming stimuli into electrical spike pulses, for multistage processing locally and throughout the brain. Such processing ultimately results in the production of a variety of perceptions, memory creation and consolidation, as well as a broad array of cognitive, motor, and other activities.

For semantic processing and knowledge representation, sets of salient signal attributes or parameters are progressively coded, bound, and integrated in networks, for extraction at higher levels (e.g., concepts and engrams). Such sets or profiles of co-occurring attributes represent the informational content of such signals in functional circuits.

In the brain, these attributes can include object and event features, action characteristics, semantic and syntactic relationships, concepts, valence, conation, affect, and memory, as embodied in concept and knowledge representation networks (MacKay, 1987; Baker et al., 1997; Klimesch, 2012).

Such attributes are encoded with different and adaptable saliences, that is the color of a banana has less salience than its identity as a kind of fruit. Learning, emotional states, etc. enable the expansion/deletion and/or modification of these attributes. Subsets of attributes can be more or less independent of each other. The color or odor of a typical banana is more closely tied to its state of ripeness than its length or heft. The brain can be regarded as an elaborate search engine that dynamically encodes and retrieves information related to any of these dimensions. Note that this is a much richer set of parameters than is utilized in any text search engine.

Common sets of such significant attributes could plausibly be embodied and maintained in recurrent reverberant circuits in cortical and subcortical structures (Kubie, 1930; Lorente de Nó and Fulton, 1949; Johnson et al., 2009). Brains can be regarded as self-organized networks of reverberant delay paths and cycles (loops). Thought to be associated with short-term memory and plasticity functions and facilitated by NMDA receptors, reverberating circuits are dynamic and maintained in a constant state of readiness through spontaneous activity, such that they are capable of rapidly changing their communications and connectivity.

As functional neural assemblies, these reverberant neural circuits are analogous to different radio channels. Both sets of entities are well-determined, and utilize precise tuning mechanisms to achieve high selectivity in communication transmissions.

A radio tuning circuit selects among the many channels picked up by its antenna, matched filters, etc., and locks onto a designated frequency channel in order to receive its signal. A key difference between radio communication systems and neural reverberant circuit networks, is that the neural circuits are also content-addressable and modifiable. The reverberant circuits can dynamically interact with each other. Except for local interference effects, radio channels do not change their contents as a consequence of interacting with each other.

### 8.3 Reverberant Circuits

A reverberant neuronal circuit can selectively lock onto particular attributes encoded by different patterns of neural activity. Combinations of attributes can be selected on the basis of more complex combinations of neural patterns. The profiles of incoming neural signals may be compared (e.g., cross-correlated) with those in existing reverberant circuits. This matching operation constitutes a content-addressable memory mechanism. With memory or knowledge circuits, one or more of these reverberant circuits are selected for activation if a close match is found. If a close, though not exact match, is found, an existing reverberant circuit may still be partially activated or be provisionally modified to incorporate additional attributes or to drop inessential features.

When no resonant match is attained for a novel neural activity pattern associated with a new kind of object or event, then a new reverberant circuit may be established to be used for future scans. This is an automatic self-organizing mechanism by which memory consolidation and learning, even with only one exemplar, (e.g., unsupervised “1-shot learning”) could be represented. The matching operations themselves are conditioned by physiological states, salience, volition, and other attentional factors. These kinds of reverberant architectures can be regarded as time- and frequency-domain analogs of the channel-coded adaptive resonance architectures that have been refined and applied to a wide array of psychological phenomena (Grossberg, 2021).

Like radio channels that are close in frequency, two or more reverberant circuits that share common features, can interfere with each other to cause the analogs of radio static: ambiguity, confusion, poor resolution, etc. More powerful channels can interfere with or dominate weaker ones (e.g., masking or blocking).

Creating new reverberant neural circuits is analogous to establishing new radio broadcast channels *de novo*, including low-powered pop-up “pirate channels.” As with memories and concepts, the process of adding channels is open-ended (Piaget, 1980; Cariani, 2012), such that there is no intrinsic limit to the number of these that can be established.

Associative networks are comprised of a set of reverberant circuits that share some attributes in common with each other. As in spreading activation networks, sufficiently activated reverberant circuits may in turn successively activate additional reverberant circuits that have attributes in common. Reverberant circuits are dynamically reinforced by repeated activation. Repeated reinforcement of these reverberant circuits increases their relative power within the network, as well as the likelihood of their subsequently triggering other matched reverberant circuits. Chains of successively triggered associative reverberant circuits could conceivably support eventual, delayed recall of memory through an ongoing, iterative search process.

Like high powered radio broadcast transmitters that reach further distances than lower powered broadcast transmitters, reverberant circuits that have been recently reinforced through high salience or reward, acquire a greater ability to activate additional associated circuits.

Relay stations, whether for radio or neuronal signals, enable the amplification and propagation of modulated signals for robust, remote, and redundant distribution.

There are many ways in which signals may interact. As discussed above regarding multiplexed codes (§5.6), neural signals from multiple modalities can be interleaved and propagated together. Similarly in the radio domain, Code Division Multiple Access (CDMA) enables multiple transmitters to send their separately coded signals over a given channel simultaneously. This technology has been broadly applied from cell phones to radar. Indeed, radio signals and communications systems themselves are fundamentally based on well-defined operations and manipulations of signal interactions.

### 8.4 Hologram-Like Operations and Representations

Ideas related to holographic representations, despite being on its margins, have a long history in theoretical neuroscience. Non-local distributed neural representations, interference networks and memory traces were envisioned by Lashley in the 1930’s and early 1940’s (Lashley, 1942, 1960; John, 1982; Pribram, 1982; Orbach, 1998; Nadel and Maurer, 2020). While working on electron microscopy in the late 1940’s, Gabor (1949) invented holograms, for which he subsequently received the Nobel Prize in Physics in 1971. Subsequently Pribram (1971, 1982), Freeman (1975), Thatcher and John (1977), John (1982), Longuet-Higgins (1987a, 1989), and others (Pietsch, 1981; Willshaw, 1981; Heinz, 2004, 2010) proposed hologram-like brain theories. Distributed, quasi-holographic channel-coded representations based on circular convolutions for support of pattern completion and compositional operations have also been proposed for artificial associative neural networks (Plate, 1995, 2003). Whereas all holographic memories are distributed, not all distributed memories are necessarily holographic in the strict sense of utilizing signal interferences based on relative phase, frequency, timing, and/or temporal pattern.

The operations required for holographic devices are fairly straightforward. The output response of a stored signal pattern is effectively an autocorrelation convolved with an incoming signal pattern [see also the optical autocorrelatograms of Meyer-Eppler in Lange (1967)]. In optical laser holography, a single coherent source signal is split into two identical signal beams, one of which, the object beam, is subsequently altered by bouncing off a physical object, and then reflected back to a common receiver (e.g., photographic plate), with the other, the reference beam, which is not. The coincident arrival of these two signal streams, like intersecting wave fronts constructively and destructively combining, create a cross correlation interference pattern containing the combined information of both signal beams in phase and amplitude relations. The new information (e.g., object image) can be decoded by convolving the interference pattern with the original signal beam.

Significantly, this interference pattern is distributed across all of the photographic plate. The entire object image can be recovered from any piece of it, albeit with lower resolution as the piece diminishes in size. Furthermore, the hologram plate can be similarly exposed to multiple scenes prior to development. Although the resolution of the hologram decreases as the number of scenes increases, each of the original scenes can be recovered by exposing the hologram to a portion of any given scene, i.e., a process of pattern completion.

Pribram (1971) neatly summed up optical holography as “an instantaneous analog cross correlation formed by matched filters,” and noted that:

“In the brain correlation can take place at various levels. In more peripheral stations correlation occurs between successive configurations produced by receptor excitation: the residuals left by adaptation by decrementing from a buffer memory register to be updated by current input. At more central stations correlation entails a more complex interaction: at any moment input is correlated not only with the configuration of excitation existing at any one locus, but also with patterns arriving from other stations.”

Although interacting signals need not be synchronous or coherent, some special objects and functions can be derived when they are. Separately, Longuet-Higgins (1969, 1987b, 1989) also championed holographic long-term memory storage and retrieval mechanisms using time-domain correlations.

Longuet-Higgins extended these principles to describe a holophone, where distributed memory can be encoded, associated, and retrieved by interference patterns created by temporal sequences. In his implementation, the holophone records a temporal signal via the variable gains from each channel of a bank of narrow bandwidth filters. These filters function as oscillators, that can later be activated, amplified, and combined for play out. Memory of the recorded signals is incorporated in the sequences of channel gains. Subsequent excitation of this filter bank by a segment of the original recording, should then enable the entire original recording to be regenerated (pattern completion).

Although positing that the cochlea may be able to meet the tight timing requirements for such a mechanism, Longuett-Higgins warns that this holophone design “must not be taken too literally,” due to noise, lack of invariance to tempo shifts, limited bandwidth, etc. He does, however, “suggest that the general principles underlying its operation should be given very serious consideration in connection with the brain” (Longuet-Higgins, 1969).

## 9. Summary

The brain is a remarkably, multifaceted complex machine. It is a dynamic self-organizing, autoencoding, change-detection, pattern correlation, network machine that takes in information and converts it to perception, memory, cognition, motor actions, and more. The neuroscience community has set for itself the daunting task of reverse-engineering it. How the brain works is one of the most fundamental unsolved problems in all of science.

Drawing on the collective knowledge of neuroscientists past and present, the consolidated “bottom-up, top-down” approach taken here focuses on key components, comparing and contrasting them to systems with similar operations, in order to point to promising avenues for further advancement.

The roles of time, neural codes, and signal processing operations are critical to understanding the functional mechanisms of the brain and nervous system. Outlined here are observed and putative neural codes, phase-locking, temporal patterns, recurrent timing networks, synchronies, oscillations, temporal coupling, the interactions of these with each other, and possible functions and roles they play within and across levels. Some experimental and methodological suggestions to focus and facilitate such research are proffered. Finally, an end-to-end systems level approach examines radio communications systems, in terms of individual components, signal processing operations, and possible relations to specific cortical functions. Recurrent timing nets, associative reverberant networks, and holographic functions are proposed as mechanisms for consideration in prospective neural architectures.

Intriguingly, the brain and these systems all share common mathematical descriptions of their operations, including time/phase coherence, logical AND/OR/NOT relations, autocorrelations, cross correlations, and convolutions. Simple signal processing operations are remarkably powerful in characterizing and emulating many functional roles in neural systems.

Going into the future, it is suggested that research should consider and explore:

(1) The roles of complex time codes and their integration at multiple levels.
(2) Autoencoding, self-organizing modulation/demodulation mechanisms.
(3) New modes of pattern generation via self-organizing, self-referential autocorrelations and cross correlations. New patterns may be subsequently combined and (de)convolved with others, both to carry feed-forward information as well as to incorporate predictive information and corrective feed-back.
(4) Associative reverberant networks for dynamic memory encoding and retrieval,
(5) Yet unforeseen possible functional roles for interacting oscillations and waves, including heterodyning analogs, for signal representation, processing, temporal coupling, and tuning,
(6) Holographic techniques that encode and recover objects in time and space by creating correlational, interference patterns of coherent signals that are split, resynchronized and compared after taking separate trajectories. Sufficiently coherent population signals in the brain, similarly split and synchronized, could each provide reference beams for the other. As with optical holograms, information can in principle be redundantly encoded and retrieved both locally and globally.

## Data Availability Statement

The original contributions presented in this study are included in the article/supplementary material, further inquiries can be directed to the corresponding author.

## Author Contributions

Both authors have made a substantial, direct, and intellectual contribution to the work, and have approved it for publication.

## Conflict of Interest

The authors declare that the research was conducted in the absence of any commercial or financial relationships that could be construed as a potential conflict of interest.

## Publisher’s Note

All claims expressed in this article are solely those of the authors and do not necessarily represent those of their affiliated organizations, or those of the publisher, the editors and the reviewers. Any product that may be evaluated in this article, or claim that may be made by its manufacturer, is not guaranteed or endorsed by the publisher.
